# Accidental poisoning in children: a single centre case series study in Bangladesh

**DOI:** 10.1136/bmjpo-2022-001541

**Published:** 2022-07-20

**Authors:** Ahsan Ahmed, Md Hasanul Banna Siam, Mohammad Shojon, Md Mahdi Hasan, Enayetur Raheem, Mohammad Sorowar Hossain

**Affiliations:** 1 Department of Emerging and Neglected Diseases, Biomedical Research Foundation, Dhaka, Bangladesh; 2 Dhaka Medical College, Dhaka, Bangladesh; 3 School of Environment and Life Sciences, Independent University, Bangladesh, Dhaka, Bangladesh

**Keywords:** Epidemiology

## Abstract

**Background:**

Accidental poisoning is a leading cause of unintentional injuries among children in low-income and middle-income countries (LMICs). The overall aspect of this unintentional poisoning is poorly understood in Bangladesh. The objectives of this study were (1) to explore the socio-demographic factors and circumstantial context of accidental poisoning and (2) the prevalence of the type of substances causing it.

**Methods:**

A descriptive case series study was conducted from April 2019 to February 2020 at a tertiary level hospital of the capital city Dhaka in Bangladesh. Children under 10 years of age admitted to the hospital with accidental poisoning were enrolled in this study. Parents of hospitalised children were interviewed face-to-face using a structured questionnaire. Descriptive statistics were used for data analysis.

**Results:**

A total of 223 children were recruited in this study. Children between 2 and 5 years (60%), men (61%) and children with agility (65.5%) were among the prevalent victims. The majority of cases occurred (65%) in a nuclear family setting. Most mothers (85%) of these children were non-working and most incidents took place in parents’ homes (~82%). Nearly 70% of the poisoning incidents took place in the presence of parents and over half of these occurred in the bedroom. Kerosene was the prevalent cause (33%) of accidental poisoning while insecticide/pesticide ranked second (26.5%) followed by medicines (17%) and household chemicals (12). In one-third (31.4%) of the cases, poisoning chemicals were stored in soft drink bottles while two-thirds (67.3%) of the cases were kept in containers other than original ones. Although over 80 parents somewhat knew that chemicals could be harmful to the children if ingested, most of them did not take the safety measures.

**Conclusion:**

In this present study we found that preschool-aged children were more victims of accidental poisoning mostly by ingesting kerosene and a majority of the incidents took place in the bedroom while parents were present at home. Our study findings would serve as a baseline for designing future intervention studies and policies.

**WHAT IS ALREADY KNOWN ON THIS TOPIC**

- Poisoning is a leading cause of morbidity and mortality as an unintentional injury all over the world which is seen preventable in many settings by taking region-specific measures.
- High-income countries have achieved significant reduction of poisoning incidents by taking definite measures but low-income and middle-income countries lag behind due to gap in knowledge and insights to prevent it.

**WHAT THIS STUDY ADDS**

- As a lower middle-income country, in-house settings of poisoning cases have been explored in this study.
- Kerosene and insecticides/pesticides were the most frequent poisoning substances.
- Poisonous chemicals often stored in soft drink bottles/containers.

**HOW THIS STUDY MIGHT AFFECT RESEARCH, PRACTICE OR POLICY**

- This study may open up the scope of extensive research into in-house settings regarding accidental poisoning.
- This study may play a baseline role to adopt significant changes in raising awareness about proper disposal of soft drink bottles and confronting soft drink companies to play role for that purpose.
- The policymakers should rethink controlling the use and bringing advanced regulatory measures regarding practice of kerosene, insecticides, pesticides and other harmful chemicals use.

## Introduction

Globally, poisoning is one of the leading causes of all unintentional injuries in children.^1^ Poisonous substances can cause significant morbidity or mortality when ingested, inhaled, injected or absorbed through the skin in a high concentration. Acute toxicity is linked with long-term neuropsychiatric health consequences.^2–4^ According to the WHO, nearly 200 000 people die each year due to accidental poisoning. Around 80% of these deaths occur in low-income and middle-income countries (LMICs). Accidental poisoning accounts for 10% of the total burden of unintentional injuries among children in LMICs.^1 5 6^ These incidents are associated with a number of factors that have been identified in different settings.^7–9^ Notably, about 15% of unintentional poisoning-related deaths occur below the age of 5 years and many cases go unreported or under-reported, or misreported.^10^

The causative factors and outcome of unintentional poisoning cases are influenced by various socio-demographic and economic factors and also by the availability and the quality of the medical facilities in any given region.^2 9 11^ Accidental poisoning has also an economic impact on the family to get medical treatment for the victim as most of the cases are from LMICs. In a study conducted in Sri Lanka, on an average US$31.83 was spent per patient for treatment purposes, with an addition of US$14.03 per patient in transport.^12^

Bangladesh is a low-middle income nation with over 30 million children under the age of 10.^13 14^ A nationwide survey in 1997–1998 estimated an incidence of 11 poisoning cases per 100 000 per year in Bangladesh.^15^ Unlike high-income countries (HICs), there is no awareness or practice of using childproof containers for poisonous substances in Bangladesh. Government directives also have not properly addressed the issue of using child-protective containers for chemical agents. Even though accidental poisoning is one of the leading causes of unintentional injury among children in LMICs, it has received little attention in Bangladesh. There is no comprehensive epidemiological study focusing household factors for accidental poisoning among children in Bangladesh.^10 16–18^ All the prior studies here had a small sample size and primarily focused on the prevalence of chemicals that caused accidental poisoning and on outcomes. Thus, we aimed to investigate the current prevalence of the type of chemical agents causing accidental poisoning among children and related socio-demographic and circumstantial context in children admitted in one of the largest tertiary level hospitals in Bangladesh.

## Methods

### Study site and sample size

This descriptive case series study was conducted from 20 April 2019 to 21 February 2020 at Dhaka Medical College Hospital, a tertiary level hospital situated at the capital city Dhaka in Bangladesh. Notably, data collection was completed before the declaration of the COVID-19 pandemic in Bangladesh (first case was reported on 8 March 2020). The hospital has one of the highest capacities to accommodate patients and the highest level of facilities for all the economic classes of the country. The admission capacity of the paediatric medicine department is approximately 30 children per day but around twice as many patients are admitted usually. Moreover, the hospital mostly serves middle to lower-income class people. This study enrolled children under 10 years of age who were admitted via the emergency department with a diagnosis of poisoning (unintentional) through convenience sampling approach. Children above 10 years of age and cases with food poisoning, adverse drug reactions, snake bite, animal venom or insect bite were excluded.

### Definition of poisoning substances

A poisonous substance was defined as any agent that had the capacity or potential to produce toxic effects or morbidity or mortality to the child while ingested, inhaled, injected or absorbed through the skin in quantities enough to cause physiological and neuropsychological effects. Thus, compounds such as pharmaceuticals, petroleum products, household chemicals, insecticides and pesticides were considered poisonous in this study.

### Questionnaire and data collection

The study questionnaire (added in online supplemental file 1) was developed based on existing literature and expert opinion (including a paediatrician and public health researcher) to understand the epidemiology and possible factors related to childhood accidental poisoning in Bangladesh.^19 20^ In the questionnaire, basic demographic information included: age of the child, parents’ age and their marital status, education, profession, monthly family income and place of living (urban/semi-urban/rural). One question was asked about the child’s agility (yes/no). Questions related to poisoning included: type of poisonous substance ingested/inhaled, time and exact place of the incidence, parents’ presence at that time, time taken to seek medical care, type of container and how chemicals and medicines were stored in the house. Parents/guardians were asked: whether they knew the substance taken by the child was harmful (yes/no), whether they alerted the child about the harmful sides of stored chemicals/medicines/kerosene (yes/no). The draft questionnaire was piloted on five parents and revised accordingly.

10.1136/bmjpo-2022-001541.supp1 Supplementary data

Face-to-face interviews with children’s attendants were conducted using a structured questionnaire in the native language (Bangla). Two of the authors (medical student) and an intern physician took part in the data collection procedure. No children were interviewed; instead, information about accidental poisoning incidents was provided by their parents or guardians.

### Statistical analysis

Data were stored and secured in Research Electronic Data Capture (REDCap) platform hosted at Biomedical Research Foundation.^21^ Descriptive statistical analysis was performed. When applicable, descriptive data were expressed as percentages and means, along with SD. Pearson’s χ^2^ test was performed to determine the association between categorical data (time of incident took place and presence of parents at home; type of poisoning and place in home where the incident took place) and a p value smaller than 0.05 was considered statistically significant.

### Ethical consideration

Each subject provided verbal and/or written informed consent at their discretion. The parents were approached first and informed about the study’s nature and objective. Following consent (oral and/or written), participants were registered for an interview. Some respondents were unable to sign their names; in these instances, the questionnaire was marked as a verbal agreement. In the context of Bangladesh, patients or patients’ attendants usually do not refuse to participate in such interview sessions in public hospitals. No patients’ attendants declined to participate in this study. For data collection, approval was taken from the head of the Paediatric Medicine Department, Dhaka Medical College Hospital. Parents were assured with good faith that their shared information would be confidential under doctor–patient relationship oath so that they could talk freely about the incident.

### Patient participation statement

Patients were involved in the design stage, questionnaire development and conduct of the research. The questionnaire was trialled with five patients and was modified as per observation and respondents’ experience. Over the period of data collection, it was ensured that the patients participate in the study when they are stable after a hectic poisoning accident. Moreover, patients’ welfare in the crowded hospital was a priority. We intend to disseminate the study results in mass public to aware them as well as to policy levels to mitigate the preventable poisoning accidents.

## Results

A total of 223 children with accidental poisoning were enrolled in the study. In 60% of cases, children were aged between 2 and 5 years and male children (61%) were the major victim. Around two-thirds (65.5%) of the children were highly active in behaviour or prompt in nature (means they were up and doing and more active than peers according to the parents) (table 1). Nearly 43% of the mothers had more than 10 years of schooling while it was 36.8% for fathers. More than 85% of mothers were stay-at-home mothers (table 1). The majority of incidents occurred in a nuclear family setting (65%). In most families, fathers were the breadwinners (83%); and the monthly incomes of a major portion of the families (79.8%) were below US$300 (table 1).

**Table 1:** Socio-demographic characteristics of parents and hospitalised children

Characteristics	N (%)	CI (95%)
Characteristics of children		
Child age group (years) (n=223)		
Median age in years (25th percentile–75th percentile)	3 (1.9–4)	
Sex (n=223)		
Male	136 (61.0)	(0.54 to 0.67)
Female	87 (39.0)	(0.33 to 0.45)
Highly active/prompt in nature (n=222)		
Yes	146 (65.5)	(0.59 to 0.71)
No	76 (34.1)	(0.28 to 0.40)
Characteristics of parents		
Father’s age (years) (n=217)		
Median age	34	
≤25	9 (4.0)	(0.02 to 0.07)
26–30	63 (28.3)	(0.23 to 0.34)
31–35	71 (31.8)	(0.26 to 0.38)
36–40	44 (19.7)	(0.15 to 0.25)
>40	36 (16.1)	(0.12 to 0.21)
Mother’s age (years) (n=219)		
Median age	28	
≤25	86 (38.6)	(0.32 to 0.45)
26–30	71 (31.8)	(0.26 to 0.38)
31–35	53 (23.8)	(0.19 to 0.30)
36–40	9 (4.0)	(0.02 to 0.07)
>40	4 (1.8)	(0.00 to 0.04)
Father’s education (n=220)		
Illiterate	24 (10.9)	(0.07 to 0.15)
Primary	29 (13.2)	(0.09 to 0.18)
Secondary	82 (36.3)	(0.31 to 0.43)
Higher secondary	54 (24.6)	(0.19 to 0.30)
Graduation	31 (14.1)	(0.09 to 0.19)
Mother’s education (n=222)		
Illiterate	28 (12.6)	(0.08 to 0.17)
Primary	45 (20.3)	(0.15 to 0.26)
Secondary	96 (43.2)	(0.37 to 0.49)
Higher secondary	32 (14.4)	(0.10 to 0.19)
Graduation	21 (9.5)	(0.06 to 0.14)
Father’s profession (n=221)		
Service	91 (41.2)	(0.34 to 0.47)
Business	68 (30.8)	(0.25 to 0.37)
Farming	12 (5.4)	(0.03 to 0.09)
Day labour	15 (6.8)	(0.04 to 0.11)
Others	35 (15.8)	(0.11 to 0.21)
Mother’s profession (n=222)		
Housewife	191 (86.0)	(0.80 to 0.89)
Service	15 (6.8)	(0.04 to 0.11)
Business	2 (0.9)	(0.00 to 0.03)
Farming	2 (0.9)	(0.00 to 0.03)
Day labour	0 (0)	(0.00 to 0.01)
Others	12 (5.4)	(0.03 to 0.09)
Monthly family income (US$) (n=223)		
<180	75 (33.6)	(0.28 to 0.40)
180–300	103 (46.2)	(0.39 to 0.53)
301–590	38 (17.0)	(0.13 to 0.22)
>590	7 (3.1)	(0.01 to 0.06)
Employment status of parents (n=222)		
Only the father works outside	186 (83.8)	(0.78 to 0.88)
Both parents work outside	32 (14.4)	(0.10 to 0.19)
Both parents stay home	1 (0.45)	(0.00 to 0.02)
Other	3 (1.4)	(0.00 to 0.04)
Residence (n=223)		
Metropolitan	97 (43.5)	(0.37 to 0.50)
Suburban	83 (37.2)	(0.31 to 0.44)
Village	43 (19.3)	(0.15 to 0.25)
Type of family (n=223)		
Nuclear	145 (65.0)	(0.58 to 0.71)
Large	78 (35.0)	(0.29 to 0.41)

Table 2 reports the circumstantial context of poisoning incidence. More than two-thirds of the incidents (82.1%) took place in parents’ homes and 57% of those were in apartment buildings. Within the house, half of the cases (51.1%) took place in bedrooms, while 14.8% occurred in the kitchen. About 30% of incidents occurred before noon while 24.2% of incidents took place at night and 16.6% in the afternoon. Over two-thirds of incidents (70.9%) occurred in the presence of at least one parent at home while nearly 30% of incidents took place when neither of the parents were at home. Interestingly, incidents during the morning were linked to both parents being outside, while night-time incidents were linked to both parents being at home (p<0.005).

**Table 2:** Circumstantial context for accidental poisoning

Characteristics	N (%)	CI (95%)
Household factors		
Type of household (n=223)		
Apartment building	127 (57.0)	(0.50 to 0.63)
House made of tin	89 (39.9)	(0.34 to 0.46)
House made of mud/wood/thatch	6 (2.7)	(0.01 to 0.06)
Slum	1 (0.4)	(0.00 to 0.02)
Where the incident took place (n=223)		
Parents’ home	183 (82.1)	(0.76 to 0.86)
Grandparents’ home	20 (9.0)	(0.05 to 0.13)
School	1 (4.0)	(0.00 to 0.02)
Other	19 (8.5)	(0.05 to 0.13)
In which part of the house the incident took place (n=223)		
Bedroom	114 (51.1)	(0.44 to 0.58)
Drawing/dining room	21 (9.4)	(0.06 to 0.14)
Kitchen	33 (14.8)	(0.10 to 0.20)
Storeroom	13 (5.8)	(0.03 to 0.09)
Yard	12 (5.4)	(0.03 to 0.09)
Other	30 (13.5)	(0.09 to 0.18)
Time when the incident took place		
Morning	65 (29.1)	(0.23 to 0.35)
Noon	67 (30.0)	(0.24 to 0.36)
Afternoon	37 (16.6)	(0.12 to 0.22)
Night	54 (24.2)	(0.19 to 0.30)
Parents’ presence during the incident (n=223)		
None	60 (26.9)	(0.21 to 0.33)
Only one	123 (55.2)	(0.48 to 0.61)
Both	40 (17.9)	(0.13 to 0.23)
Time taken to bring the child to medical attention (n=223)		
Less than 2 hours	150 (67.3)	(0.61 to 0.73)
2–4 hours	54 (24.2)	(0.19 to 0.30)
4–6 hours	15 (6.7)	(0.04 to 0.11)
More than 6 hours	4 (1.8)	(0.00 to 0.04)
Safety issues		
The poison was in its original container (n=223)		
No	150 (67.3)	(0.61 to 0.73)
Yes	73 (32.7)	(0.27 to 0.39)
Chemical stored in soft-drinks’ bottles (n=223)		
No	153 (68.6)	(0.62 to 0.74)
Yes	70 (31.4)	(0.25 to 0.38)
Chemical/medicine stored in an unsafe place at home (n=223)		
No	64 (28.7)	(0.23 to 0.35)
Yes	159 (71.3)	(0.65 to 0.77)
Event of poisoning happened earlier with the children or with siblings (n=223)		
No	220 (98.7)	(0.96 to 0.99)
Yes	3 (1.3)	(0.00 to 0.04)

Ingestion of kerosene was caused in nearly one-third (32.7%) of hospitalised cases, with insecticides/pesticides being the second most common cause (26.5%). Poisoning from ingestion of medicines was found to occur in 17.5% of the cases, whereas poisoning due to household chemicals such as bleach/toiletries/phenyl occurred in 11.7% of the cases. Other substances were responsible for 11.7% of the cases. Type of poisoning was found to be associated with the place in home the incident took place (p<0.005) (table 3).

**Table 3:** Cross-tabulation between the type of poisoning and the place in home the incident took place in

Type of poisoning	Place in home where incidents took place						
	Bedroom	Drawing/dining room	Kitchen	Store room	Yard	Other	Total
Kerosene	28 (38.4%)	28 (38.4%)	16 (21.9%)	4 (5.5%)	7 (9.6%)	10 (13.7%)	73 (100%)
Insecticide/pesticide	28 (47.5%)	6 (10.2%)	11 (18.6%)	7 (11.9%)	3 (5.1%)	4 (6.8%)	59 (100%)
Medicines	35 (89.7%)	2 (5.1%)	0 (0.0%)	0 (0.0%)	0 (0.0%)	2 (5.1%)	39 (100%)
Household chemicals (bleach/toiletries/phenyl, etc)	12 (46.2%)	4 (15.4%)	3 (11.5%)	1 (3.8%)	0 (0.0%)	6 (23.1%)	26 (100%)
Other	11 (42.3%)	1 (3.8%)	3 (11.5%)	1 (3.8%)	2 (7.7%)	8 (30.8%)	26 (100%)
Total	114 (51.1%)	21 (9.4%)	33 (14.8%)	13 (5.8%)	12 (5.4%)	30 (13.5%)	223 (100%)

X^2^=50.48; df=20; p=0.0002; Fisher’s exact test p=0.00099

As for the storage of chemical/medicine, about 71.3% of the respondents reported that the chemical/medicine substances were kept in places within the child’s reach. In one-third of the cases (31.4%), chemicals such as kerosene were stored in bottles of soft drinks.

Almost all incidents (98.7%) took place for the first time in the family. About 20% of parents were unaware whether ingested substances were harmful. Most parents (78.4%) did not take protective measures for storing chemicals at home. Two-thirds of the children (67.3%) were taken to medical attention within 2 hours, while 24.2% were within 2–4 hours and 8.5% in more than 4 hours. More than half of the children (53.8%) stayed at the hospital for 1 day, while 39.5% of them stayed for 2 days (online supplemental figure 1). The mean hospital stay was 1.8 days (SD: 2.42).

10.1136/bmjpo-2022-001541.supp2 Supplementary data

## Discussion

To the best of our knowledge, this is the first comprehensive study from Bangladesh to understand the prevalence and characteristics of the cases of unintentional poisoning in children. We found that kerosene was the most prevalent poisoning agent and a majority of the incidents took place in the bedrooms while parents were present at home.

In South Asian countries, kerosene remains the most prevalent cause of accidental poisoning among children. Our study finding is also consistent with previous studies from this region.^2 20 22 23^ In contrast, household chemicals and medicine are the main causes of children’s accidental poisoning in HICs.^2–4 11 24^ Bangladesh consumes 100 000 tons of kerosene a year.^25^ This is mainly used for cooking and lightening in shanties in the metropolitans, peri-urban and rural areas where there is no or limited service for natural gas. Most hospitalised children in the present study came from underdeveloped (shanties and peri-urban) areas. In nearly all kerosene poisoning cases, liquid hydrocarbon was stored in soft drink bottles (plastic). As a consequence, children are deceived to think of kerosene as soft drinks. A similar practice is also reported from some other South Asian countries.^20 26^ Single-use plastic bottles/containers, especially soft drinks bottles are repeatedly used in LMICs for storing food and liquids. Given the popularity of soft drinks particularly Coca Cola, Pepsi, Sprite and other local ones, storing kerosene or liquid chemicals in these empty soft drink bottles is very common in day-to-day life here. Therefore, popular soft drinks companies should also contribute to raise community-level awareness to prevent accidental poisoning in children, particularly in LMICs.

Despite 85% of mothers being housewives (stay-at-home), over 70% of accidental incidents occurred when parents were at home. Similar to prior studies in LMICs, most mothers were younger,^27–30^ and nearly all incidents occurred for the first time in the family. Similar to other studies, preschool-aged male children were more prone to accidental poisoning.^31–35^ Lack of experience in parenting and not taking practical measures might contribute to these incidents. Even though most parents were aware of the potential accident, they were not able to translate their knowledge into practice. This psychological paradox in this region warrants further study.

The home environmental factors might have served as barriers to proper safety practice since bedrooms were the the most common place where half of the incidents took place in our study. However, prior studies reported kitchens and yards were the prevalent locations for accidental poisoning.^36^ In our study, most poisoning cases were from disadvantaged families (lower economic stratum) who were mostly living in small apartment houses with only one or two small rooms. Due to lack of enough space, all of the daily chores and commodities are kept and surrounded in a crowded manner in these small houses. Moreover, Bangladeshi families hardly practice store room keeping and usually store substances near to hand. Because of the exploratory nature of children, poisonous substances easily find their way into the hands of children. Effective parental (mother-centric) awareness and the use of child-protective containers could be viable solutions for preventing unintentional childhood poisoning.

### Limitations

A large sample and in-house context exploration are the strengths of this study. However, most childhood poisoning cases were from metropolitan or nearby areas. Therefore, our study did not portray the scenario of rural areas where nearly 70% of the Bangladeshi population live. Our findings also may not be generalisable to other urban areas of the country as Dhaka city is the most crowded megacity in the world.^37^ However, our study findings could be useful for similarly crowded megacities in LMICs. In Bangladesh, patients with a lower economic background generally seek healthcare services in public hospitals and thus our findings are not representative of all economic classes. Moreover, since we used a convenience sampling approach for enrolling patients, this may introduce a bias in the outcome and therefore our findings should be interpreted with caution.

## Conclusion

In this present study, we found that kerosene was the most prevalent causative agent of poisoning among children and a majority of the incidents took place in the bedroom while parents were present at home. Our study findings would serve as a baseline for designing future intervention studies (such as mother-centric prevention) and policies to make soft drink companies socially responsible and could also enforce the issue of child protective containers.

## Supplementary Material

Author's manuscript

## Data Availability

All data relevant to the study are included in the article or uploaded as supplementary information.
