# Supplementary material for: Accidental poisoning in children: a single centre case series study in Bangladesh
Source: BMJ Paediatr Open. 2022 Jul 20;6(1):e001541. doi: 10.1136/bmjpo-2022-001541 (PMC9305806; doi:10.1136/bmjpo-2022-001541)
Supplement: Supplementary data [file bmjpo-2022-001541supp001.pdf]

Questionnaire

1. Name of children: ____________________________________ **2.** Age: ________year
3. Sex: ☐ Male ☐ Female
4. Father's age_________
5. Mother's age_________
6. Address: ____
7. Living place: ☐ Metropolitan ☐ Sub-urban ☐ Village
8. Father's highest education: ☐ uneducated ☐ Primary ☐ Secondary ☐ Higher secondary ☐ Graduate
9. Father's profession: ☐ Service holder ☐ Businessman ☐ Farmer ☐ Day-laborer ☐ Others
10. Mother's highest education: ☐ uneducated ☐ Primary ☐ Secondary ☐ Higher secondary ☐ Graduate
11. Mother's profession: ☐ Housewife ☐ Service holder ☐ Businesswoman ☐ Farmer ☐ Day-laborer ☐ Others

12. Parents' marital status:
☐ Married and living together
☐ Married but living separately (husband/wife may live abroad)
☐ Divorced ☐ Separated ☐ N/A
13. Family type:
☐ Nuclear
☐ Extended
14. Family members: ___________
15. Monthly income of family: ☐ <15,000 ☐ 15,000-24,999 ☐ 25,000-49,999 ☐ >50,000
16. Type of household ☐ Apartment ☐ House made of tin
☐ House made of mud/wood/thatch ☐ Slum
17. Parents' employment status:
☐ Only father works outside ☐ Both parent work outside
☐ Both parents stay home ☐ Others (please mention)

18. Is the child agile in nature? ☐ Yes ☐ No
19. Did such poisoning incident happen earlier with the children? ☐ Yes ☐ No
If yes, how many times? _______
20. Did such poisoning incident happen earlier with the sibiling(s)? ☐ Yes ☐ No
21. Type of poisoning:
☐ Medicine
☐ Kerosene
☐ Household chemical **(**bleach/toiletries/phenyl etc.**)**
☐ Insecticide/pesticide
☐ Others _________________

22. Where in home the incident did take place?
☐ Bedroom
☐ Drawing/dining room

Name of data collector: Date:

Questionnaire

- ☐ Kitchen
- ☐ Godown (store-house)
- ☐ House-yard
- ☐ Others

23. Which home did the incident take place**?**
- ☐ Parents' home
- ☐ Grandparents' home
- ☐ School
- ☐ Others

24.when did it happen**? (**morning/noon/afternoon/night**)**

25. Time of first consultation with doctor since incidence:
- ☐ Less than 2 hours
- ☐ 2-4 hours
- ☐ 4-6 hours
- ☐ More than 6 hours

26. Who were at home during the incident? (if needed mark multiple)
- ☐ mother
- ☐ father
- ☐ elder sibling(s)
- ☐ younger sibling(s)
- ☐ relative
- ☐ house-help
- ☐ others
- ☐ none

27. Type of container of the poisonous substance**?**
- ☐ No container
- ☐ Original container where it supposed to be
- ☐ Some other container where it supposed not to be

28. Storage practice of medicine and chemicals in the household? (if applicable, multiple answers can be ticked)
- ☐ Chemical stored in locked place**?**
- ☐ Chemical stored in higher place**?**
- ☐ Medicine stored in locked place**?**
- ☐ Medicine stored in higher place**?**

29. Availability of harmful substances**?**
- ☐ Kerosene/chemical/medicine stored in soft-drinks bottles
- ☐ Chemical/medicine stored in safe place
- ☐ Chemical/medicine stored in unsafe place

30. How many days the children had to stay at the hospital for treatment? _________days

31. How much it costed for the treatment of the children (hospital, medicine)? _______taka

32. Other costs apart from treatment (transport,stipends of parents' stay etc.)?_______taka

33. Did you know the substance was harmful**? (**Yes/no**)**

34. Did you ever warn the child about the potential harmful substances at home? (Yes/no)

35. Do you think that you could prevent the incident**? (**yes/no/don't know**)**

Name of data collector: Date:

Questionnaire

36. Do you think that you will be able to protect such possible incidents in future**?**
... (The respondent will opine freely. It is important)

Name of data collector: Date:
