# Supplementary figures and images for: Accidental poisoning in children: a single centre case series study in Bangladesh
Source: BMJ Paediatr Open. 2022 Jul 20;6(1):e001541. doi: 10.1136/bmjpo-2022-001541 (PMC9305806; doi:10.1136/bmjpo-2022-001541)

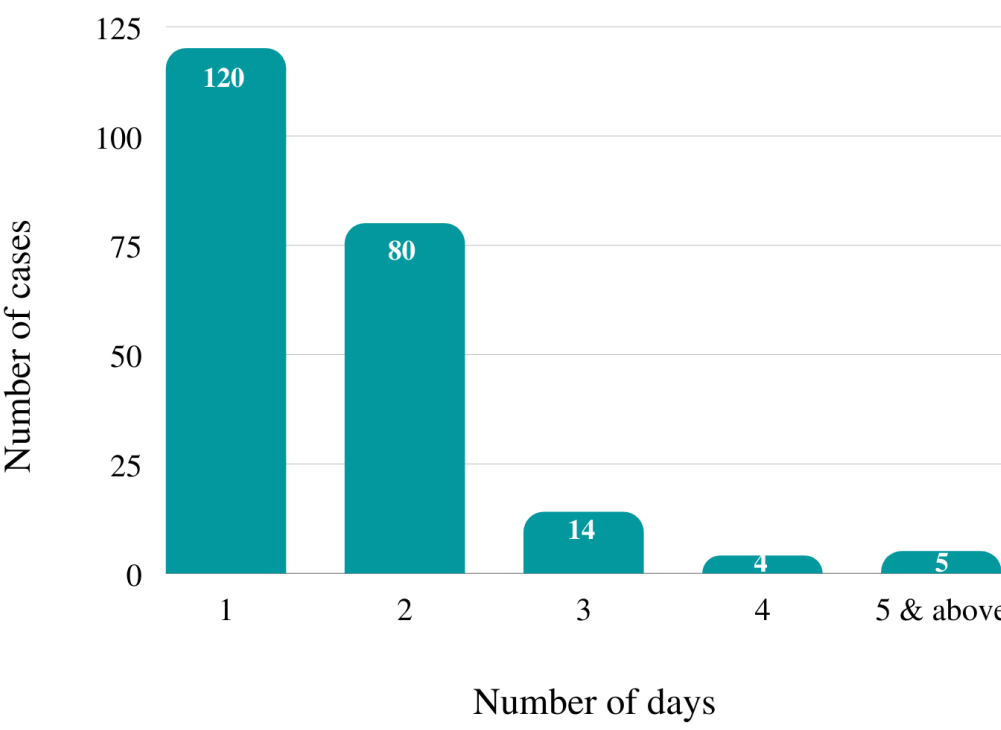

Supplement: Supplementary data [file bmjpo-2022-001541supp002.pdf]
