# Supplementary material for: Accidental poisoning in children: a single centre case series study in Bangladesh
Source: BMJ Paediatr Open. 2022 Jul 20;6(1):e001541. doi: 10.1136/bmjpo-2022-001541 (PMC9305806; doi:10.1136/bmjpo-2022-001541)
Supplement: Author's manuscript [file bmjpo-2022-001541.draft_revisions.pdf]

BMJ Paediatrics Open

BMJ Paediatrics Open is committed to open peer review. As part of this commitment we make the peer review history of every article we publish publicly available.

When an article is published we post the peer reviewers’ comments and the authors’ responses online. We also post the versions of the paper that were used during peer review. These are the versions that the peer review comments apply to.

The versions of the paper that follow are the versions that were submitted during the peer review process. They are not the versions of record or the final published versions. They should not be cited or distributed as the published version of this manuscript.

BMJ Paediatrics Open is an open access journal and the full, final, typeset and author-corrected version of record of the manuscript is available on our site with no access controls, subscription charges or pay-per-view fees (http://bmjpaedsopen.bmj.com).

If you have any questions on BMJ Paediatrics Open’s open peer review process please email info.bmjpo@bmj.com

BMJ Paediatrics Open

# Factors affecting accidental poisoning among children in Bangladesh– Lessons learned from an observational study at a tertiary level hospital, Dhaka

| | |
|---|---|
| Journal: | *BMJ Paediatrics Open* |
| Manuscript ID | bmjpo-2022-001541 |
| Article Type: | Original research |
| Date Submitted by the Author: | 11-May-2022 |
| Complete List of Authors: | Ahmed, Ahsan; Biomedical Research Foundation; Dhaka Medical College<br>Hasanul Banna, Md.; Biomedical Research Foundation<br>Shojon, Mohammad; Dhaka Medical College<br>Mahdi Hasan, Md.; Biomedical Research Foundation<br>Raheem, Enayetur; Biomedical Research Foundation<br>Hossain, Mohammad; Biomedical Research Foundation |
| Keywords: | Epidemiology |
| | |

SCHOLARONE™
Manuscripts

BMJ

*I, the Submitting Author has the right to grant and does grant on behalf of all authors of the Work (as defined in the below author licence), an exclusive licence and/or a non-exclusive licence for contributions from authors who are: i) UK Crown employees; ii) where BMJ has agreed a CC-BY licence shall apply, and/or iii) in accordance with the terms applicable for US Federal Government officers or employees acting as part of their official duties; on a worldwide, perpetual, irrevocable, royalty-free basis to BMJ Publishing Group Ltd ("BMJ") its licensees and where the relevant Journal is co-owned by BMJ to the co-owners of the Journal, to publish the Work in this journal and any other BMJ products and to exploit all rights, as set out in our licence.*

*The Submitting Author accepts and understands that any supply made under these terms is made by BMJ to the Submitting Author unless you are acting as an employee on behalf of your employer or a postgraduate student of an affiliated institution which is paying any applicable article publishing charge ("APC") for Open Access articles. Where the Submitting Author wishes to make the Work available on an Open Access basis (and intends to pay the relevant APC), the terms of reuse of such Open Access shall be governed by a Creative Commons licence – details of these licences and which Creative Commons licence will apply to this Work are set out in our licence referred to above.*

*Other than as permitted in any relevant BMJ Author's Self Archiving Policies, I confirm this Work has not been accepted for publication elsewhere, is not being considered for publication elsewhere and does not duplicate material already published. I confirm all authors consent to publication of this Work and authorise the granting of this licence.*

Confidential: For Review Only

# Factors affecting accidental poisoning among children in Bangladesh– Lessons learned from an observational study at a tertiary level hospital, Dhaka

Ahsan Ahmed[1, 2], Md. Hasanul Banna Siam[1], Mohammad Shojon[2], Md. Mahdi Hasan[1], Enayetur Raheem[1] and Mohammad Sorowar Hossain[1’3]

1 Department of Emerging and Neglected Diseases, Biomedical Research Foundation, Dhaka, Bangladesh

[2]Dhaka Medical College, Dhaka, Bangladesh

[3]School of Environment and Life Sciences, Independent University, Bangladesh, Dhaka, Bangladesh

*** Correspondence:**

Mohammad Sorowar Hossain

Email: sorowar.hossain@brfbd.org

**Keywords:** Injury, Prevention, Epidemiology, Poisoning, Children, Dhaka, Bangladesh

Confidential: For Review Only

# Abstract

## Background

Accidental poisoning is a leading cause of unintentional injuries among children in developing countries. The overall aspect of this unintentional poisoning is poorly understood in Bangladesh. The objectives of this study were 1) to explore the socio-demographic factors and circumstantial context of accidental poisoning and 2) the prevalence of the type of substances causing it.

## Methods

A cross-sectional prospective study was conducted from April, 2019 to February, 2020 at a tertiary level hospital of the capital city Dhaka in Bangladesh. Children under 10 years of age admitted to the hospital with accidental poisoning were enrolled in this study. Parents of hospitalized children were interviewed face-to-face using a structured questionnaire. Descriptive statistics were used for data analysis.

## Results

A total of 223 children were recruited in this study. Children between 2 to 5 years (60%), male (61%), and children with agile (65.5%) were among the prevalent victims. The majority of cases occurred (65%) in a nuclear family setting. Most mothers (85%) of these children were non-working and most incidents took place in parents' homes (~82%). Nearly 70% of the poisoning incidents took place in the presence of parents and over half of these occurred in the bedroom. Kerosene was the prevalent cause (33%) of accidental poisoning while insecticide/pesticide ranked second (26.5%) followed by medicines (17%) and household chemicals (12). In one-third (31.4%) cases, poisoning chemicals were stored in soft-drinks bottles while two-thirds (67.3%) were kept in containers other than original ones. Although over 80 parents somewhat knew that chemicals could be harmful to the children if ingested, most of them did not take the safety measures.


Confidential: For Review Only

## Conclusion

In this present study we found that preschool-aged children were more victims of accidental poisoning mostly by ingesting kerosene and a majority of the incidents took place in the bedroom while parents were present at home. Our study findings would serve as a baseline for designing future intervention studies and policies.

## What is already known on this subject

- Poisoning is a leading cause of morbidity and mortality as an unintentional injury all over the world which is seen preventable in many settings by taking region-specific measures.
- Developed countries have achieved significant reduction of poisoning incidents by taking definite measures but low- and middle-income countries lag behind due to gap in knowledge and insights to prevent it.

## What this study adds

- As an underdeveloped country, in-house settings leading to poisoning has been explored in this study.
- Parental factors and behaviours can play a vital role in such settings.

## How this study might affect research, practice or policy

- This study may open up scopes of extensive research into in-house settings regarding accidental poisoning.
- This study may play a baseline role to adopt significant change in parents' behaviors related to unintentional harm to their children.
- The policymakers should rethink of controlling the use and bringing advanced regulatory measures regarding kerosene, insecticides, pesticides and other harmful chemicals.



Confidential: For Review Only

## Introduction

Globally, poisoning is one of the leading causes of all unintentional injuries in children [1]. Poisons substances can cause significant morbidity or mortality when ingested, inhaled, injected, or absorbed through the skin in a high concentration. Acute toxicity is linked with long-term neuropsychiatric health consequences [2–4]. According to the World Health Organization, nearly 200,000 people die each year due to accidental poisoning. Around 80% of these deaths occur in low- and middle-income countries (LMICs) [1,5,6]. Accidental poisoning accounts for 10% of the total burden of unintentional injuries among children in developing countries [7–9]. Notably, about 15% of unintentional poisoning-related deaths occur below the age of 5 years and many cases go unreported or under-reported, or misreported [10].

The causative factors and outcome of unintentional poisoning cases are influenced by various socio-demographic and economic factors and also by the availability and the quality of the medical facilities in any given region [2,9,11]. Accidental poisoning has also an economic impact on the family to get medical treatment for the victim as most of the cases are from LMICs. In a study conducted in Sri Lanka, on an average 31.83 USD was spent per patient for treatment purposes, with an addition of 14.03 USD per patient in transport [12].

Bangladesh is a low-middle income nation with over 30 million children under the age of 10 [13,14]. A nationwide survey in 1997-1998 estimated an incidence of 11 poisoning cases per 100,000 per year in Bangladesh [15]. Unlike developed countries, there is no awareness or practice of using child-proof containers for poisonous substances in Bangladesh. Government directives also have not properly addressed the issue of using child-protective containers for chemical agents. Even though accidental poisoning is one of the leading causes of unintentional injury among children in developing countries, it has received little attention in Bangladesh. There is no comprehensive epidemiological study focusing household factors for accidental poisoning among children in Bangladesh [10,16–18]. All the prior studies here had a small sample size and primarily focused on the prevalence type of chemicals that caused accidental poisoning and on outcomes. Thus, we aimed to investigate the current prevalence of the type of chemical agents causing accidental poisoning among children and related socio-demographic and circumstantial context in children under the age of 10 in Bangladesh.





Confidential: For Review Only

# Methods

### *Study site and sample size*

This cross-sectional study was conducted from 20 April, 2019 to 21 February, 2020 at Dhaka Medical College Hospital, a tertiary level hospital situated at the capital city Dhaka in Bangladesh. Notably, data collection was completed before the declaration of the COVID-19 pandemic in Bangladesh (first case was reported in 8 March 2020). The hospital has one of the highest capacities to accommodate patients and the highest level of facilities for all the economic classes of the country. The admission capacity of the pediatric medicine department is approximately 30 children per day but around twice more patients is admitted usually. Moreover, the hospital mostly serves middle to lower-income class people.

This study recruited children under 10 years of age who were only admitted through the emergency department with a diagnosis of poisoning (unintentional) by the duty doctors at the emergency facility or attending physicians at the ward. Children above 10 years of age and cases with food poisoning, adverse drug reactions, snake bite, animal venom, or insect bite were excluded.

### *Definition of poisoning substances*

A poisonous substance was defined as any agent that had the capacity or potential to produce toxic effects or morbidity or mortality to the child while ingested, inhaled, injected, or absorbed through the skin in quantities enough to cause physiological and neuropsychological effects. Thus, compounds such as pharmaceuticals, petroleum products, household chemicals, insecticides, and pesticides were considered poisonous in this study.

### *Questionnaire and data collection*

The study questionnaire (added in supplementary materials) was developed based on existing literature and expert opinion (including a pediatrician and public health researcher) to understand the epidemiology and possible factors related to childhood accidental poisoning in Bangladesh [19,20]. In the questionnaire, basic demographic information included: age of the child, parents' age and their marital status, education, profession, monthly family income, place of living (urban/semi-urban/rural). One question was asked about the child's agility (yes/no). Questions



Confidential: For Review Only

related to poisoning included: type of poisonous substance ingested/inhaled, time and exact place of the incidence, parents' presence at that time, time taken to seek medical care, type of container, and how chemicals and medicines were stored in the house. Parents/guardians were asked: whether they knew the substance taken by the child was harmful (yes/no), whether they alerted the child about the harmful sides of stored chemicals/medicines/kerosene (yes/no). The draft questionnaire was piloted on 5 parents and revised accordingly.

Face-to-face interviews with attendants of children were conducted using a structured questionnaire in the native language (Bangla). Two of the authors (medical student) and an intern physician took part in the data collection procedure. No children were interviewed; instead, information about accidental poisoning incidents was provided by their parents or guardians.

### *Statistical analysis*

Data were stored and secured in Research Electronic Data Capture (REDCap) platform hosted at BRF [21]. Descriptive statistical analysis was performed using R statistical software. When applicable, descriptive data were expressed as percentages and means, along with standard deviation. Pearson's chi-square test was performed to determine the association between categorical data and a p-value smaller than 0.05 was considered statistically significant.

### *Ethical consideration*

The study protocol was approved by the institutional review board of Biomedical Research Foundation, Bangladesh (Ref. no: BRF/ERB/2019/004). Each subject provided verbal and/or written informed consent at their discretion. The parents were approached first and informed about the study's nature and objective. Following consent (oral and/or written), participants were registered for an interview. Some respondents were unable to sign their names; in these instances, the questionnaire was marked as a verbal agreement. For data collection, approval was taken from the head of the pediatric medicine department, Dhaka Medical College Hospital. Parents were assured with good faith that their shared information will be confidential under doctor-patient relationship oath so that they can talk freely on the incident.



Confidential: For Review Only

***Patient participation statement***

Patients were involved in the design stage, questionnaire development and conduct of the research. The questionnaire was trialled with 5 patients and was modified as per observation and respondents' experience. Over the period of data collection, it was ensured that the patients participate in the study when they are stable after a hectic poisoning accident. Moreover, patients' welfare in the crowded hospital was a priority. We intend to disseminate the study results in mass public to aware them as well as to policy levels to mitigate the preventable poisoning accidents.

## Results

A total of 223 children with accidental poisoning were enrolled in the study. Accidental poisoning was prevalent among children aged between 2 and 5 years (60%) and in male children (61%). Around two-thirds (65.5%) of the children were highly active in behaviour or prompt in nature (means they were up and doing and more active than peers according to the parents) (**Table 1**). Nearly 43% of the mother had more than 10 years of schooling while it was 36.8% for fathers. More than 85% of mothers were stay-at-home mothers (**Table 1**). The majority of incidents occurred in a nuclear family setting (65%). In most families, fathers were the bread-winner (83%); and the monthly incomes of a major portion of the families (~80%) were below USD 300 (**Table 1**).

**Figure 1** reports on the type of chemicals causing accidental poisoning. Ingestion of kerosene caused in nearly one-third (33%) hospitalized cases, with insecticides/pesticides being the second most common cause (26.5%). Poisoning from ingestion of medicines was found to occur in 17% of cases, whereas poisoning due to household chemicals such as bleach/toiletries/phenyl occurred in 12% of cases. Type of poisoning was found to associated with the place in home the incident took place ($p<0.005$) (**table 3**).

**Table 2** reports the circumstantial context of poisoning incidence. More than two-thirds of the incidents (82.1%) took place in parents' homes and only 9% in the grandparents' homes and 57% of those were apartment buildings. Within the house, half of the cases (51.1%) took place in bedrooms, while 14.8% occurred in the kitchen. About 30% of incidents occurred before noon while 24.2% of incidents took place at night and 16.6% in the afternoon. Over two-thirds of





Confidential: For Review Only

incidents (~73%) occurred in presence of at least one parent at home while nearly 27% of incidents took place when none of the parents were at home. Interestingly, incidents during the morning were linked to both parents being outside, while nighttime incidents were linked to both parents being at home ($p<0.005$).

As for the storage of chemical/medicine, about 71.3% of the respondents reported that the chemical/medicine substances were kept in places within the child's reach. In one-third of the cases (31.4%), chemicals such as kerosene were stored in bottles of soft drinks.

Almost all incidents (~99%) took place for the first time in the family. About 20% of parents were unaware whether ingested substances were harmful. Most parents (78.4%) did not take protective measures for storing chemicals at home (**Suppl. Figure 1**). Two-thirds of the children (~67%) were taken to medical attention within 2 hours, while 24.2% were within 2-4 hours and 8.5% in more than 4 hours. More than half the children (53.8%) stayed at the hospital for a day, while 39.5% of them for 2 days (**Suppl. Figure 2**). The mean hospital stay was 1.8 days (SD: 2.42).

## Discussion

To the best of our knowledge, this is the first comprehensive study from Bangladesh to understand the circumstantial factors related to unintentional poisoning in children. We found that kerosene was the most prevalent poisoning agent and a majority of the incidents took place in the bedrooms while parents were present at home.

In South Asian countries, kerosene remains the most prevalent cause of accidental poisoning among children. Our study finding is also consistent with previous studies from this region [2,20,22,23]. In contrast, household chemicals and medicine are the main causes of children's accidental poisoning in developed countries [2–4,11,24]. Bangladesh consumes 100,000 tons of kerosene a year [25]. This is mainly used for cooking and lightening in shanties in the metropolitans, peri-urban and rural areas where there is no or limited service for natural gas. Most hospitalized children in the present study came from underdeveloped (shanties and peri-urban) areas. In nearly all kerosene poisoning cases, liquid hydrocarbon was stored in soft drink bottles (plastic). As a consequence, children are deceived to think of kerosene as soft drinks. A similar practice is also reported from some other South Asian countries [20,26]. Single-use plastic

Confidential: For Review Only

bottles/containers, especially soft drinks bottles are repeatedly used in developing countries for storing food and liquids. Given the popularity of soft drinks particularly Coca Cola, Pepsi, Sprite and other local ones, storing kerosene or liquid chemicals in these empty soft drink bottles is very common in day-to-day life here. Therefore, popular soft drinks companies should also contribute to raise community-level awareness to prevent accidental poisoning in children, particularly in developing countries.

Despite 85% of mothers being housewives (stay-at-home), over 70% of accidental incidents occurred when parents were at home. Similar to prior studies in developing countries, most mothers were younger [27–30], and nearly all incidents occurred for the first time in the family. Similar to other studies, preschool aged male children were more prone to accidental poisoning [31–35]. Lack of experience in parenting and not taking practical measures might contribute to these incidents. Even though most parents were aware of the potential accident, they were not able to translate their knowledge into practice. This psychological paradox in this region warrants further study.

The home environmental factors might have served as barriers to proper safety practice since bedrooms were the commonest place where half of the incidents took place in our study. However, prior studies reported kitchens and yards were the prevalent locations for accidental poisoning [36]. In our study, most poisoning cases were from disadvantaged families (lower economic stratum) who were mostly living in small apartment houses with only one or two small rooms. Due to lack of enough space, all the daily chores and commodities are kept and surrounded in a crowded manner in these small houses. Moreover, Bangladeshi families hardly practice store room keeping and usually store substances near to hand. Because of the exploratory nature of children, poisonous substances easily find their way into the hands of children. Effective parental (mother-centric) awareness and the use of child protective containers could be a viable solution for preventing unintentional childhood poisoning.

## Limitations

A large sample and in-house context exploration are the strengths of this study. However, most childhood poisoning cases were from metropolitan or nearby areas. Therefore, our study did not portray the scenario of rural areas where nearly 70% of the Bangladeshi population lives there. Our findings also may not be generalizable to other urban areas of the country as the Dhaka city)



Confidential: For Review Only

is the most crowded megacity in the  varies broadly given Dhaka is one of the biggest megacities in the world [37]. However, our study findings could be useful for similar crowded megacities in low and middle-income countries.

## Conclusion

In this present study, we found that kerosene was the most prevalent causative agent of poisoning among children and a majority of the incidents took place in the bedroom while parents were present at home. Our study findings would serve as a baseline for designing future intervention studies and policies

## Declarations

### Funding

This research received no specific grant from any funding agency in the public, commercial or not-for-profit sectors.

### Competing interests

The authors declare that there is no conflict of interest regarding the publication of this article.

### Author contributions

MSH conceived the idea. MSH and AA designed and developed the questionnaire. ER designed the study. AA led the data collection stage along with MS. ER along with MMH analyzed data. AA, MSH, MHBS, MS, ER, MMH contributed in the result preparation. AA prepared the draft and MHBS and MSH reviewed and revised it.

### Acknowledgement

The authors would like to impart their immense gratitude to Dr. Golam Moktadir for contributing and all the doctors and nurses of the pediatric medicine department of Dhaka Medical College for being supportive at the data collection stage. The authors are especially grateful to Prof. Sayeeda Anwar, the then head of the pediatric medicine department of Dhaka Medical College Hospital



Confidential: For Review Only

and Prof. Zohora Jameela Khan, the head of the pediatric hematology and oncology department of Dhaka Medical College Hospital for providing required assistance needed at the study site.

**Patient consent form**

It is not applicable for the study as no information of any identifiable individual is disclosed.

**Authors' information**

Affiliations

*Biomedical Research Foundation, Dhaka-1230, Bangladesh*

Ahsan Ahmed, Md. Hasanul Banna Siam, Md. Mahdi Hasan, Enayetur Raheem

*Dhaka Medical College, Dhaka, Bangladesh*

Ahsan Ahmed, Mohammad Sojon.

## References

1 Chandran A, Hyder AA, Peek-Asa C. The global burden of unintentional injuries and an agenda for progress. *Epidemiol Rev* 2010;**32**:110–20. doi:10.1093/epirev/mxq009

2 Özdemir R, Bayrakci B, Tekşam Ö, *et al.* Thirty-three-year experience on childhood poisoning. *Turk J Pediatr* 2012.

3 Haghighat M, Moravej H, Moatamedi M. Epidemiology of Pediatric Acute Poisoning in Southern Iran: A Hospital-Based Study. *Bull Emerg trauma* 2013.

4 Rajka T, Heyerdahl F, Hovda KE, *et al.* Acute child poisonings in Oslo: a 2-year prospective study. *Acta Paediatr* 2007;**96**:1355–9. doi:10.1111/j.1651-2227.2007.00420.x

5 De Presgrave RF, Camacho LAB, Boas MHSV. A profile of unintentional poisoning caused by household cleaning products, disinfectants and pesticides. *Cad Saude Publica* Published Online First: 2008. doi:10.1590/S0102-311X2008001200019





Confidential: For Review Only

6 Dayasiri MBKC, Jayamanne SF, Jayasinghe CY. Risk Factors for Acute Unintentional Poisoning among Children Aged 1–5 Years in the Rural Community of Sri Lanka. *Int J Pediatr* Published Online First: 2017. doi:10.1155/2017/4375987

7 Schmertmann M, Williamson A, Black D, *et al.* Risk factors for unintentional poisoning in children aged 1-3 years in NSW Australia: A case-control study. *BMC Pediatr* Published Online First: 2013. doi:10.1186/1471-2431-13-88

8 Schmertmann M, Williamson A, Black D. Unintentional poisoning in young children: Does developmental stage predict the type of substance accessed and ingested? *Child Care Health Dev* 2014;**40**:50–9. doi:10.1111/j.1365-2214.2012.01424.x

9 Schwebel DC, Evans WD, Hoeffler SE, *et al.* Unintentional child poisoning risk: A review of causal factors and prevention studies. Child. Heal. Care. 2017. doi:10.1080/02739615.2015.1124775

10 Anwar S, Akmn R, A HSK, *et al.* Clinical Profile of Kerosene Poisoning in a Tertiary Level Hospital in Bangladesh. 2014;**38**:11–4.

11 Seif E, Gomaa R, Eisa M. A retrospective study of acute poisoning in children under 5 years old admitted to Alexandria Poison Center in Egypt. *World J Prev Med* 2016.

12 Wickramasinghe K, Steele P, Dawson A, *et al.* Cost to government health-care services of treating acute self-poisonings in a rural district in Sri Lanka. *Bull World Health Organ* 2009;**87**:180–5. doi:10.2471/BLT.08.051920

13 *Bangladesh Bureau of Statistics (BBS) Statistics and Informatics Division (SID) Ministry of Planning Government of the People's Republic of Bangladesh*. 2013. www.bbs.gov.bd (accessed 13 Feb 2021).

14 BBS. Population & Housing Census-2011. *Bangladesh Bur Stat* 2014.

15 Taylor P, Rahman F, Ali Y, *et al.* Injury Control and Safety Promotion Epidemiology of injury : Results from injury registration at a district level hospital in Bangladesh – implications for prevention in low-income countries Epidemiology of injury : Results from injury registration at a d. 2010;:37–41. doi:10.1076/icsp.8.1.29.3367

Confidential: For Review Only

16 Rashid MM, Hasan MA, Chowdhury FR. Childhood acute poisoning in a tertiary medical college hospital of Bangladesh. *Mymensingh Med J* 2007;**16**.

17 Ahmed F, Rahman MM, Tawfiq M. Clinical profile and outcomes of childhood poisoning. *Bangabandhu Sheikh Mujib Med Univ J* 2017;**10**:264. doi:10.3329/bsmmuj.v10i4.34868

18 He S, Lunnen JC, Puvanachandra P, *et al.* Global Childhood Unintentional Injury Study: Multisite Surveillance Data. *Am J Public Health* 2014;**104**:e79–84. doi:10.2105/AJPH.2013.301607

19 Hyder AA, Wali S, Fishman S, *et al.* The burden of unintentional injuries among the under-five population in South Asia. *Acta Paediatr* 2008;**97**:267–75. doi:10.1111/j.1651-2227.2008.00670.x

20 Ahmed B, Fatmi Z, Siddiqui AR, *et al.* Predictors of unintentional poisoning among children under 5 years of age in karachi: A matched case-control study. *Inj Prev* 2011;**17**:27–33. doi:10.1136/ip.2010.027524

21 Harris PA, Taylor R, Thielke R, *et al.* Research electronic data capture (REDCap)-A metadata-driven methodology and workflow process for providing translational research informatics support. *J Biomed Inform* 2009;**42**:377–81. doi:10.1016/j.jbi.2008.08.010

22 Kohli U, Kuttiat VS, Lodha R, *et al.* Profile of childhood poisoning at a tertiary care centre in north India. *Indian J Pediatr* Published Online First: 2008. doi:10.1007/s12098-008-0105-7

23 Childhood Accidental Poisoning in Tafila. *Child Accid Poisoning Tafila* 2007.

24 Pawłowicz U, Wasilewska A, Olańnski W, *et al.* Epidemiological study of acute poisoning in children: A 5-year retrospective study in the Paediatric University Hospital in Białystok, Poland. *Emerg Med J* Published Online First: 2013. doi:10.1136/emermed-2012-201376

25 Diesel, kerosene prices 23pc up | The Daily Star. https://www.thedailystar.net/news/bangladesh/news/diesel-kerosene-prices-23pc-2221876 (accessed 18 Mar 2022).

Confidential: For Review Only

26 Yaqoob M, Yar M, Farooq M, *et al.* Acute Poisoning in Children : Etiological Agents , Risk Factors and Outcomes. 2007;**1**:42–4.

27 Alazab RM, Elmougy MT, Fayad RA, *et al.* Risk factors of acute poisoning among children: A study at a poisoning unit of a university hospital in Egypt. *South East Asia J Public Heal* 2013;**2**:41–7. doi:10.3329/seajph.v2i2.15943

28 Abbas SK, Tikmani SS, Siddiqui NT. Accidental poisoning in children. *J Pak Med Assoc* 2012;**62**:331–4.

29 Fatmi Z, Kazi A, Hadden WC, *et al.* Incidence and pattern of unintentional injuries and resulting disability among children under 5 years of age: Results of the National Health Survey of Pakistan. *Paediatr Perinat Epidemiol* 2009;**23**:229–38. doi:10.1111/j.1365-3016.2009.01024.x

30 Reimers A, Laflamme L. Neighbourhood social and socio-economic composition and injury risks. *Acta Paediatr Int J Paediatr* 2005;**94**:1488–94. doi:10.1080/08035250510038929

31 Tarvadi PV, Bakkannavar SM, Palimar V, *et al.* Gender Differences in Childhood Poisoning. *J Heal Allied Sci NU* 2013;**03**:069–72. doi:10.1055/s-0040-1703657

32 Jan MA, Siddiqui TS, Ahmed N, *et al.* Mushroom poisoning in children: clinical presentation and outcome. *J Ayub Med Coll Abbottabad* 2008;**20**:99–101.

33 Gauvin F, Bailey B, Bratton SL. Hospitalizations for pediatric intoxication in Washington State, 1987-1997. *Arch Pediatr Adolesc Med* 2001;**155**:1105–10. doi:10.1001/archpedi.155.10.1105

34 Reith DM, Pitt WR, Hockey R. Childhood poisoning in Queensland: An analysis of presentation and admission rates. *J Paediatr Child Health* 2001;**37**:446–50. doi:10.1046/j.1440-1754.2001.00666.x

35 Injuries in the European Union: Summary 2003-2005 - Portail Santé // Grand-Duché de Luxembourg. https://sante.public.lu/fr/publications/i/injuries-eu-stat-2003-2005/index.html (accessed 19 Mar 2022).

Confidential: For Review Only

36 Dayasiri MBKC, Jayamanne SF, Jayasinghe CY. Patterns and outcome of acute poisoning among children in rural Sri Lanka. *BMC Pediatr* 2018;**18**:1–8. doi:10.1186/s12887-018-1246-0

37 Hossain MS. Megacity-centric mass mobility during Eid holidays: a unique concern for infectious disease transmission in Bangladesh. *Trop Med Health* 2022;**50**:4–6. doi:10.1186/s41182-022-00417-4

Confidential: For Review Only

**Table 1: Socio-demographic characteristics of parents and hospitalized children**

| Characteristics | N (%) |
|---|---|
| ***Characteristics of children*** | |
| **Child age group (years) (n=223)** | |
| Median age in years (IQR) | 3 (2.1) |
| **Sex (n=223)** | |
| Male | 136 (61.0) |
| Female | 87 (39.0) |
| **Highly active/prompt in nature (n=222)** | |
| Yes | 146 (65.5) |
| No | 76 (34.1) |
| ***Characteristics of parents*** | |
| **Father's age (years) (n=217)** | |
| Median age | 34 |
| ≤25 | 9 (4.0) |
| 26-40 | 63 (28.3) |
| 31-35 | 71 (31.8) |
| 36-40 | 44 (19.7) |
| >40 | 36 (16.1) |
| **Mother's age (years) (n=219)** | |
| Median age | 28 |
| ≤25 | 86 (38.6) |
| 26-30 | 71 (31.8) |
| 31-35 | 53 (23.8) |
| 36-40 | 9 (4.0) |
| >40 | 4 (1.8) |

Confidential: For Review Only

| | |
|---|---|
| **Father's education (n=220)** | |
| Illiterate | 24 (10.9) |
| Primary | 29 (13.2) |
| Secondary | 82 (36.3) |
| Higher secondary | 54 (24.6) |
| Graduation | 31 (14.1) |
| **Mother's education (n=222)** | |
| Illiterate | 28 (12.6) |
| Primary | 45 (20.3) |
| Secondary | 96 (43.2) |
| Higher secondary | 32 (14.4) |
| Graduation | 21 (9.5) |
| **Father's profession (n=221)** | |
| Service | 91 (41.2) |
| Business | 68 (30.8) |
| Farming | 12 (5.4) |
| Day labor | 15 (6.8) |
| Others | 35 (15.8) |
| **Mother's profession (n=222)** | |
| Housewife | 191 (86.0) |
| Service | 15 (6.8) |
| Business | 2 (0.9) |
| Farming | 2 (0.9) |
| Day labor | 0 (0) |
| Others | 12 (5.4) |
| **Monthly family income (USD) (n=223)** | |
| <180 | 75 (33.6) |
| 180-300 | 103 (46.2) |
| 301-590 | 38 (17.0) |
| >590 | 7 (3.1) |
| **Employment status of parents (n=222)** | |

Confidential: For Review Only

| | |
|---|---|
| Only the father works outside | 186 (83.8) |
| Both parents work outside | 32 (14.4) |
| Both parents stay home | 1 (0.45) |
| Other | 3 (1.4) |
| **Residence (n=223)** | |
| Metropolitan | 97 (43.5) |
| Suburban | 83 (37.2) |
| Village | 43 (19.3) |
| **Type of family (n=223)** | |
| Nuclear | 145 (65.0) |
| Large | 78 (35.0) |

**Table 2: Circumstantial context for accidental poisoning**

| **Characteristics** | **N (%)** |
|---|---|
| ***Household factors*** | |
| **Type of household (n=223)** | |
| Apartment building | 127 (57.0) |
| House made of Tin | 89 (39.9) |
| House made of mud/wood/thatch | 6 (2.7) |
| Slum | 1 (0.4) |
| **Where the incident took place- (n=223)** | |
| Parents home | 183 (82.1) |
| Grandparents' home | 20 (9.0) |
| School | 1 (4.0) |
| Other | 19 (8.5) |
| **In which part of the house the incident took place- (n=223)** | |
| Bedroom | 114 (51.1) |
| Drawing/Dining Room | 21 (9.4) |

Confidential: For Review Only

| | |
|---|---|
| Kitchen | 33 (14.8) |
| Storeroom | 13 (5.8) |
| Yard | 12 (5.4) |
| Other | 30 (13.5) |
| **When the incident took place- (n=223)** | |
| Morning | 65 (29.1) |
| Noon | 67 (30.0) |
| Afternoon | 37 (16.6) |
| Night | 54 (24.2) |
| **Parents' presence during the incident- (n=223)** | |
| None | 60 (26.9) |
| Only one | 123 (52.2) |
| Both | 40 (17.9) |
| **Time taken to bring the child to medical attention- (n=223)** | |
| Less than 2 hours | 150 (67.3) |
| 2-4 hours | 54 (24.2) |
| 4-6 hours | 15 (6.7) |
| More than 6 hours | 4 (1.8) |
| ***Safety issues*** | |
| **The poison was in its original container- (n=223)** | |
| No | 150 (67.3) |
| Yes | 73 (32.7) |
| **Chemical stored in soft-drinks' bottles- (n=223)** | |
| No | 153 (68.6) |
| Yes | 70 (31.4) |
| **Chemical/medicine stored in an unsafe place at home- (n=223)** | |
| No | 64 (28.7) |
| Yes | 159 (71.3) |
| **Event of poisoning happened earlier with the children or with siblings- (n=223)** | |



Confidential: For Review Only

| | |
|---|---|
| No | 220 (98.7) |
| Yes | 3 (1.3) |

**Table 3: Cross-tabulation between type of poisoning and the place in home the incident took place in**

| **Type of poisoning** | **Place in home where incidents took place** | | | | | | |
|---|---|---|---|---|---|---|---|
| | Bedroom | Drawing /Dining room | Kitchen | Store room | Yard | Other | **Total** |
| Medicines | 35 (89.7%) | 2 (5.1%) | 0 (0.0%) | 0 (0.0%) | 0 (0.0%) | 2 (5.1%) | 39 (100%) |
| Kerosene | 28 (38.4%) | 8 (11.0%) | 16 (21.9%) | 4 (5.5%) | 7 (9.6%) | 10 (13.7%) | 73 (100%) |
| Household chemicals (bleach/toiletries/phenyl etc.) | 12 (46.2%) | 4 (15.4%) | 3 (11.5%) | 1 (3.8%) | 0 (0.0%) | 6 (23.1%) | 26 (100%) |
| Insecticide/ Pesticide | 28 (47.5%) | 6 (10.2%) | 11 (18.6%) | 7 (11.9%) | 3 (5.1%) | 4 (6.8%) | 59 (100%) |
| Other | 11 (42.3%) | 1 (3.8%) | 3 (11.5%) | 1 (3.8%) | 2 (7.7%) | 8 (30.8%) | 26 (100%) |
| **Total** | 114 (51.1%) | 21 (9.4%) | 33 (14.8%) | 13 (5.8%) | 12 (5.4%) | 30 (13.5%) | **223 (100%)** |
| $X^2 = 50.48$; $df = 20$; $p = 0.0002$; Fisher's Exact test p = 0.00099 | | | | | | | |

## Figures

Figure 1: Distribution of types of poisoning

Confidential: For Review Only

## Supplementary files

Suppl. Figure 1: Awareness level of the parents

Suppl. Figure 2: The duration of hospital stays

The questionnaire of the study

The STROBE list of the cross-sectional study

Confidential: For Review Only

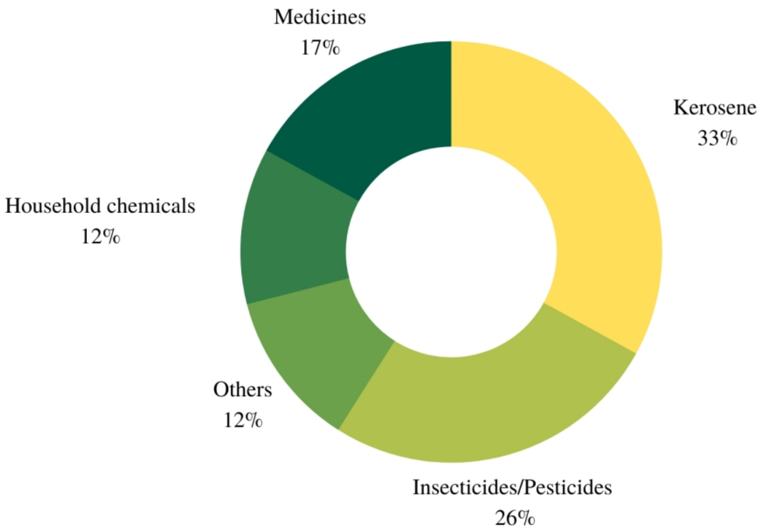


413x269mm (118 x 118 DPI)

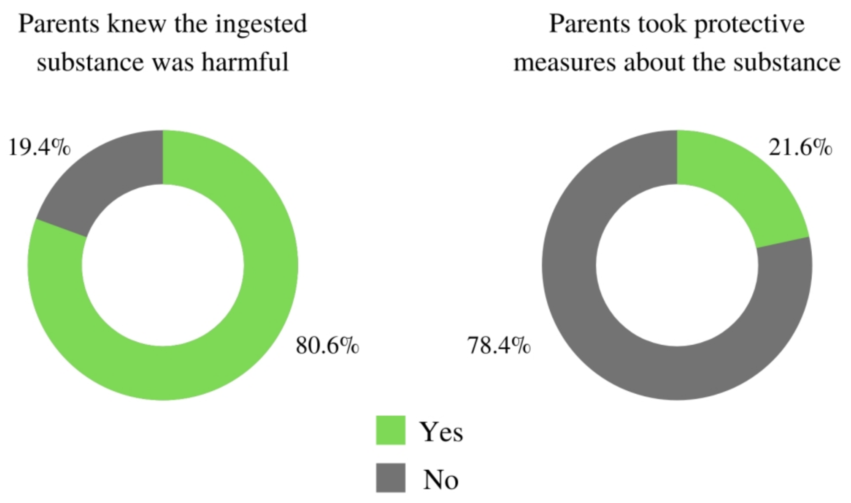


413x269mm (118 x 118 DPI)

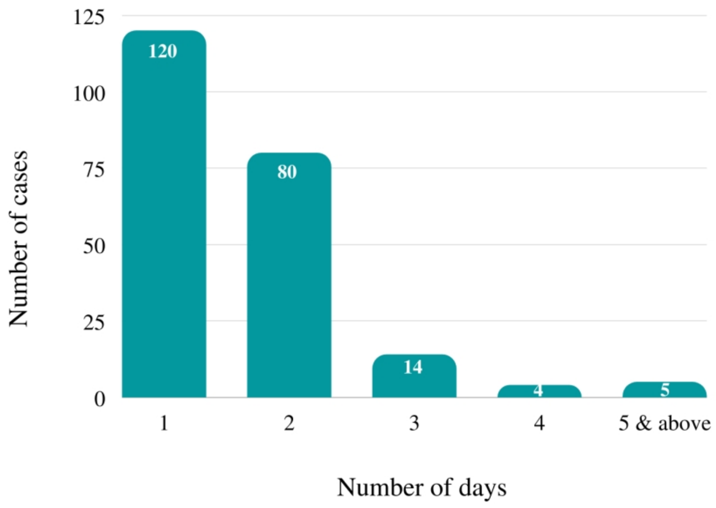


413x269mm (118 x 118 DPI)

BMJ Paediatrics Open

# Accidental poisoning in children—a single centre case series study in Bangladesh

| | |
|---|---|
| Journal: | *BMJ Paediatrics Open* |
| Manuscript ID | bmjpo-2022-001541.R1 |
| Article Type: | Original research |
| Date Submitted by the Author: | 08-Jun-2022 |
| Complete List of Authors: | Ahmed, Ahsan; Biomedical Research Foundation; Dhaka Medical College<br>Hasanul Banna, Md.; Biomedical Research Foundation<br>Shojon, Mohammad; Dhaka Medical College<br>Mahdi Hasan, Md.; Biomedical Research Foundation<br>Raheem, Enayetur; Biomedical Research Foundation<br>Hossain, Mohammad; Biomedical Research Foundation |
| Keywords: | Epidemiology |
| | |

SCHOLARONE™
Manuscripts

*I, the Submitting Author has the right to grant and does grant on behalf of all authors of the Work (as defined in the below author licence), an exclusive licence and/or a non-exclusive licence for contributions from authors who are: i) UK Crown employees; ii) where BMJ has agreed a CC-BY licence shall apply, and/or iii) in accordance with the terms applicable for US Federal Government officers or employees acting as part of their official duties; on a worldwide, perpetual, irrevocable, royalty-free basis to BMJ Publishing Group Ltd ("BMJ") its licensees and where the relevant Journal is co-owned by BMJ to the co-owners of the Journal, to publish the Work in this journal and any other BMJ products and to exploit all rights, as set out in our licence.*

*The Submitting Author accepts and understands that any supply made under these terms is made by BMJ to the Submitting Author unless you are acting as an employee on behalf of your employer or a postgraduate student of an affiliated institution which is paying any applicable article publishing charge ("APC") for Open Access articles. Where the Submitting Author wishes to make the Work available on an Open Access basis (and intends to pay the relevant APC), the terms of reuse of such Open Access shall be governed by a Creative Commons licence – details of these licences and which Creative Commons licence will apply to this Work are set out in our licence referred to above.*

*Other than as permitted in any relevant BMJ Author's Self Archiving Policies, I confirm this Work has not been accepted for publication elsewhere, is not being considered for publication elsewhere and does not duplicate material already published. I confirm all authors consent to publication of this Work and authorise the granting of this licence.*

Confidential: For Review Only

# Accidental poisoning in children—a single centre case series study in Bangladesh

Ahsan Ahmed[1, 2], Md. Hasanul Banna Siam[1], Mohammad Shojon[2], Md. Mahdi Hasan[1], Enayetur Raheem[1] and Mohammad Sorowar Hossain[1,3]

1 Department of Emerging and Neglected Diseases, Biomedical Research Foundation, Dhaka, Bangladesh

[2]Dhaka Medical College, Dhaka, Bangladesh

[3]School of Environment and Life Sciences, Independent University, Bangladesh, Dhaka, Bangladesh

*** Correspondence:**

Mohammad Sorowar Hossain

Email: sorowar.hossain@brfbd.org

**Keywords:** Injury, Prevention, Epidemiology, Poisoning, Children, Dhaka, Bangladesh

Confidential: For Review Only

# Abstract

## Background

Accidental poisoning is a leading cause of unintentional injuries among children in developing countries. The overall aspect of this unintentional poisoning is poorly understood in Bangladesh. The objectives of this study were 1) to explore the socio-demographic factors and circumstantial context of accidental poisoning and 2) the prevalence of the type of substances causing it.

## Methods

A descriptive case series study was conducted from April, 2019 to February, 2020 at a tertiary level hospital of the capital city Dhaka in Bangladesh. Children under 10 years of age admitted to the hospital with accidental poisoning were enrolled in this study. Parents of hospitalized children were interviewed face-to-face using a structured questionnaire. Descriptive statistics were used for data analysis.

## Results

A total of 223 children were recruited in this study. Children between 2 to 5 years (60%), male (61%), and children with agile (65.5%) were among the prevalent victims. The majority of cases occurred (65%) in a nuclear family setting. Most mothers (85%) of these children were non-working and most incidents took place in parents' homes (~82%). Nearly 70% of the poisoning incidents took place in the presence of parents and over half of these occurred in the bedroom. Kerosene was the prevalent cause (33%) of accidental poisoning while insecticide/pesticide ranked second (26.5%) followed by medicines (17%) and household chemicals (12). In one-third (31.4%) cases, poisoning chemicals were stored in soft-drinks bottles while two-thirds (67.3%) were kept in containers other than original ones. Although over 80 parents somewhat knew that chemicals could be harmful to the children if ingested, most of them did not take the safety measures.


Confidential: For Review Only

**Conclusion**

In this present study we found that preschool-aged children were more victims of accidental poisoning mostly by ingesting kerosene and a majority of the incidents took place in the bedroom while parents were present at home. Our study findings would serve as a baseline for designing future intervention studies and policies.

## What is already known on this subject

- Poisoning is a leading cause of morbidity and mortality as an unintentional injury all over the world which is seen preventable in many settings by taking region-specific measures.
- Developed countries have achieved significant reduction of poisoning incidents by taking definite measures but low- and middle-income countries lag behind due to gap in knowledge and insights to prevent it.

## What this study adds

- As an underdeveloped country, in-house settings of poisoning cases has been explored in this study.
- Kerosene and insecticides/pesticides were the most frequent poisoning substances.
  Poisonous chemicals often stored in soft drink bottles/containers.

## How this study might affect research, practice or policy

- This study may open up the scope of extensive research into in-house settings regarding accidental poisoning.
- This study may play a baseline role to adopt significant changes in raising awareness about proper disposal of soft drinks bottles and confronting soft-drink companies to play role for that purpose. .
- The policymakers should rethink controlling the use and bringing advanced regulatory measures regarding practice of kerosene, insecticides, pesticides and other harmful chemicals use.

Confidential: For Review Only

# Introduction

Globally, poisoning is one of the leading causes of all unintentional injuries in children [1]. Poisonous substances can cause significant morbidity or mortality when ingested, inhaled, injected, or absorbed through the skin in a high concentration. Acute toxicity is linked with long-term neuropsychiatric health consequences [2–4]. According to the World Health Organization, nearly 200,000 people die each year due to accidental poisoning. Around 80% of these deaths occur in low- and middle-income countries (LMICs). Accidental poisoning accounts for 10% of the total burden of unintentional injuries among children in developing countries [1,5,6]. These incidents are associated with a number of factors that have been identified in different settings [7–9]. Notably, about 15% of unintentional poisoning-related deaths occur below the age of 5 years and many cases go unreported or under-reported, or misreported [10].

The causative factors and outcome of unintentional poisoning cases are influenced by various socio-demographic and economic factors and also by the availability and the quality of the medical facilities in any given region [2,9,11]. Accidental poisoning has also an economic impact on the family to get medical treatment for the victim as most of the cases are from LMICs. In a study conducted in Sri Lanka, on an average 31.83 USD was spent per patient for treatment purposes, with an addition of 14.03 USD per patient in transport [12].

Bangladesh is a low-middle income nation with over 30 million children under the age of 10 [13,14]. A nationwide survey in 1997-1998 estimated an incidence of 11 poisoning cases per 100,000 per year in Bangladesh [15]. Unlike developed countries, there is no awareness or practice of using child-proof containers for poisonous substances in Bangladesh. Government directives also have not properly addressed the issue of using child-protective containers for chemical agents. Even though accidental poisoning is one of the leading causes of unintentional injury among children in developing countries, it has received little attention in Bangladesh. There is no comprehensive epidemiological study focusing household factors for accidental poisoning among children in Bangladesh [10,16–18]. All the prior studies here had a small sample size and primarily focused on the prevalence of chemicals that caused accidental poisoning and on outcomes. Thus, we aimed to investigate the current prevalence of the type of chemical agents causing accidental

Confidential: For Review Only

poisoning among children and related socio-demographic and circumstantial context in children admitted in one of the largest tertiary level hospitals in Bangladesh.

## Methods

***Study site and sample size***

This descriptive case series studyl study was conducted from 20 April, 2019 to 21 February, 2020 at Dhaka Medical College Hospital, a tertiary level hospital situated at the capital city Dhaka in Bangladesh. Notably, data collection was completed before the declaration of the COVID-19 pandemic in Bangladesh (first case was reported on 8 March 2020). The hospital has one of the highest capacities to accommodate patients and the highest level of facilities for all the economic classes of the country. The admission capacity of the pediatric medicine department is approximately 30 children per day but around twice as many patients are admitted usually. Moreover, the hospital mostly serves middle to lower-income class people.

This study recruited children under 10 years of age who were only admitted through the emergency department with a diagnosis of poisoning (unintentional) by the duty doctors at the emergency facility or attending physicians at the ward. Children above 10 years of age and cases with food poisoning, adverse drug reactions, snake bite, animal venom, or insect bite were excluded.

***Definition of poisoning substances***

A poisonous substance was defined as any agent that had the capacity or potential to produce toxic effects or morbidity or mortality to the child while ingested, inhaled, injected, or absorbed through the skin in quantities enough to cause physiological and neuropsychological effects. Thus, compounds such as pharmaceuticals, petroleum products, household chemicals, insecticides, and pesticides were considered poisonous in this study.

***Questionnaire and data collection***

The study questionnaire (added in supplementary materials) was developed based on existing literature and expert opinion (including a pediatrician and public health researcher) to understand the epidemiology and possible factors related to childhood accidental poisoning in Bangladesh [19,20]. In the questionnaire, basic demographic information included: age of the child, parents' age and their marital status, education, profession, monthly family income, place of living



Confidential: For Review Only

(urban/semi-urban/rural). One question was asked about the child's agility (yes/no). Questions related to poisoning included: type of poisonous substance ingested/inhaled, time and exact place of the incidence, parents' presence at that time, time taken to seek medical care, type of container, and how chemicals and medicines were stored in the house. Parents/guardians were asked: whether they knew the substance taken by the child was harmful (yes/no), whether they alerted the child about the harmful sides of stored chemicals/medicines/kerosene (yes/no). The draft questionnaire was piloted on 5 parents and revised accordingly.

Face-to-face interviews with children's attendants were conducted using a structured questionnaire in the native language (Bangla). Two of the authors (medical student) and an intern physician took part in the data collection procedure. No children were interviewed; instead, information about accidental poisoning incidents was provided by their parents or guardians.

***Statistical analysis***

Data were stored and secured in Research Electronic Data Capture (REDCap) platform hosted at BRF [21]. Descriptive statistical analysis was performed . When applicable, descriptive data were expressed as percentages and means, along with standard deviation. Pearson's chi-square test was performed to determine the association between categorical data (time of incident took place and presence of parents at home; type of poisoning and place in home where the incident took place) and a p-value smaller than 0.05 was considered statistically significant.

***Ethical consideration***

The study protocol was approved by the institutional review board of Biomedical Research Foundation, Bangladesh (Ref. no: BRF/ERB/2019/004). Each subject provided verbal and/or written informed consent at their discretion. The parents were approached first and informed about the study's nature and objective. Following consent (oral and/or written), participants were registered for an interview. Some respondents were unable to sign their names; in these instances, the questionnaire was marked as a verbal agreement. In the context of Bangladesh, patients or patients' attendants usually do not refuse to participate in such interview sessions in public hospitals. No patients attendants declined to participate in this study. . For data collection, approval was taken from the head of the pediatric medicine department, Dhaka Medical College Hospital.

Confidential: For Review Only

Parents were assured with good faith that their shared information would be confidential under doctor-patient relationship oath so that they could talk freely about the incident.

***Patient participation statement***

Patients were involved in the design stage, questionnaire development and conduct of the research. The questionnaire was trialled with 5 patients and was modified as per observation and respondents' experience. Over the period of data collection, it was ensured that the patients participate in the study when they are stable after a hectic poisoning accident. Moreover, patients' welfare in the crowded hospital was a priority. We intend to disseminate the study results in mass public to aware them as well as to policy levels to mitigate the preventable poisoning accidents.

## Results

A total of 223 children with accidental poisoning were enrolled in the study. . In 60% of cases, children were aged between 2 and 5 yearsand male children (61%) were the major victim. Around two-thirds (65.5%) of the children were highly active in behavior or prompt in nature (means they were up and doing and more active than peers according to the parents) (**Table 1**). Nearly 43% of the mothers had more than 10 years of schooling while it was 36.8% for fathers. More than 85% of mothers were stay-at-home mothers (**Table 1**). The majority of incidents occurred in a nuclear family setting (65%). In most families, fathers were the bread-winner (83%); and the monthly incomes of a major portion of the families (79.8%) were below USD 300 (**Table 1**).

**Table 2** reports the circumstantial context of poisoning incidence. More than two-thirds of the incidents (82.1%) took place in parents' homes and only 9% in the grandparents' homes and 57% of those were apartment buildings. Within the house, half of the cases (51.1%) took place in bedrooms, while 14.8% occurred in the kitchen. About 30% of incidents occurred before noon while 24.2% of incidents took place at night and 16.6% in the afternoon. Over two-thirds of incidents (70.9%) occurred in the presence of at least one parent at home while nearly 30% of incidents took place when neither of the parents were at home. Interestingly, incidents during the morning were linked to both parents being outside, while nighttime incidents were linked to both parents being at home ($p<0.005$).

Ingestion of kerosene was caused in nearly one-third (32.7%) of hospitalized cases, with insecticides/pesticides being the second most common cause (26.5%). Poisoning from ingestion





Confidential: For Review Only

of medicines was found to occur in 17.5% of cases, whereas poisoning due to household chemicals such as bleach/toiletries/phenyl occurred in 11.7% of cases. Other substances were responsible for 11.7% of the cases. Type of poisoning was found to associated with the place in home the incident took place ($p<0.005$) (**table 3**).

As for the storage of chemical/medicine, about 71.3% of the respondents reported that the chemical/medicine substances were kept in places within the child's reach. In one-third of the cases (31.4%), chemicals such as kerosene were stored in bottles of soft drinks.

Almost all incidents (98.7%) took place for the first time in the family. About 20% of parents were unaware whether ingested substances were harmful. Most parents (78.4%) did not take protective measures for storing chemicals at home. Two-thirds of the children (67.3%) were taken to medical attention within 2 hours, while 24.2% were within 2-4 hours and 8.5% in more than 4 hours. More than half the children (53.8%) stayed at the hospital for a day, while 39.5% of them stayed for 2 days (**Suppl. Figure 1**). The mean hospital stay was 1.8 days (SD: 2.42).

## Discussion

To the best of our knowledge, this is the first comprehensive study from Bangladesh to understand the prevalence and characteristics of the cases of unintentional poisoning in children. We found that kerosene was the most prevalent poisoning agent and a majority of the incidents took place in the bedrooms while parents were present at home.

In South Asian countries, kerosene remains the most prevalent cause of accidental poisoning among children. Our study finding is also consistent with previous studies from this region [2,20,22,23]. In contrast, household chemicals and medicine are the main causes of children's accidental poisoning in developed countries [2–4,11,24]. Bangladesh consumes 100,000 tons of kerosene a year [25]. This is mainly used for cooking and lightening in shanties in the metropolitans, peri-urban and rural areas where there is no or limited service for natural gas. Most hospitalized children in the present study came from underdeveloped (shanties and peri-urban) areas. In nearly all kerosene poisoning cases, liquid hydrocarbon was stored in soft drink bottles (plastic). As a consequence, children are deceived to think of kerosene as soft drinks. A similar practice is also reported from some other South Asian countries [20,26]. Single-use plastic bottles/containers, especially soft drinks bottles are repeatedly used in developing countries for

Confidential: For Review Only

storing food and liquids. Given the popularity of soft drinks particularly Coca Cola, Pepsi, Sprite and other local ones, storing kerosene or liquid chemicals in these empty soft drink bottles is very common in day-to-day life here. Therefore, popular soft drinks companies should also contribute to raise community-level awareness to prevent accidental poisoning in children, particularly in developing countries.

Despite 85% of mothers being housewives (stay-at-home), over 70% of accidental incidents occurred when parents were at home. Similar to prior studies in developing countries, most mothers were younger [27–30], and nearly all incidents occurred for the first time in the family. Similar to other studies, preschool aged male children were more prone to accidental poisoning [31–35]. Lack of experience in parenting and not taking practical measures might contribute to these incidents. Even though most parents were aware of the potential accident, they were not able to translate their knowledge into practice. This psychological paradox in this region warrants further study.

The home environmental factors might have served as barriers to proper safety practice since bedrooms were the commonest place where half of the incidents took place in our study. However, prior studies reported kitchens and yards were the prevalent locations for accidental poisoning [36]. In our study, most poisoning cases were from disadvantaged families (lower economic stratum) who were mostly living in small apartment houses with only one or two small rooms. Due to lack of enough space, all the daily chores and commodities are kept and surrounded in a crowded manner in these small houses. Moreover, Bangladeshi families hardly practice store room keeping and usually store substances near to hand. Because of the exploratory nature of children, poisonous substances easily find their way into the hands of children. Effective parental (mother-centric) awareness and the use of child protective containers could be a viable solution for preventing unintentional childhood poisoning.

## Limitations

A large sample and in-house context exploration are the strengths of this study. However, most childhood poisoning cases were from metropolitan or nearby areas. Therefore, our study did not portray the scenario of rural areas where nearly 70% of the Bangladeshi population lives there. Our findings also may not be generalizable to other urban areas of the country as the Dhaka city) is the most crowded megacity in the varies broadly given Dhaka is one of the biggest megacities

Confidential: For Review Only

in the world [37]. However, our study findings could be useful for similar crowded megacities in low and middle-income countries.

## Conclusion

In this present study, we found that kerosene was the most prevalent causative agent of poisoning among children and a majority of the incidents took place in the bedroom while parents were present at home. Our study findings would serve as a baseline for designing future intervention studies (such as mother centric prevention) and policies to make soft-drink compaines socially responsible and could also enforce the issue of child protective containers.

## Declarations


### Funding

This research received no specific grant from any funding agency in the public, commercial or not-for-profit sectors.


### Competing interests

The authors declare that there is no conflict of interest regarding the publication of this article.

### Author contributions

MSH conceived the idea. MSH and AA designed and developed the questionnaire. ER designed the study. AA led the data collection stage along with MS. ER along with MMH analyzed data. AA, MSH, MHBS, MS, ER, MMH contributed in the result preparation. AA prepared the draft and MHBS and MSH reviewed and revised it.


### Acknowledgement

The authors would like to impart their immense gratitude to Dr. Golam Moktadir for contributing and all the doctors and nurses of the pediatric medicine department of Dhaka Medical College for being supportive at the data collection stage. The authors are especially grateful to Prof. Sayeeda Anwar, the then head of the pediatric medicine department of Dhaka Medical College Hospital




Confidential: For Review Only

and Prof. Zohora Jameela Khan, the head of the pediatric hematology and oncology department of Dhaka Medical College Hospital for providing required assistance needed at the study site.

**Patient consent form**

It is not applicable for the study as no information of any identifiable individual is disclosed.

**Authors' information**

Affiliations

*Biomedical Research Foundation, Dhaka-1230, Bangladesh*

Ahsan Ahmed, Md. Hasanul Banna Siam, Md. Mahdi Hasan, Enayetur Raheem

*Dhaka Medical College, Dhaka, Bangladesh*

Ahsan Ahmed, Mohammad Sojon.

## References

1 Chandran A, Hyder AA, Peek-Asa C. The global burden of unintentional injuries and an agenda for progress. *Epidemiol Rev* 2010;**32**:110–20. doi:10.1093/epirev/mxq009

2 Özdemir R, Bayrakci B, Tekşam Ö, *et al.* Thirty-three-year experience on childhood poisoning. *Turk J Pediatr* 2012.

3 Haghighat M, Moravej H, Moatamedi M. Epidemiology of Pediatric Acute Poisoning in Southern Iran: A Hospital-Based Study. *Bull Emerg trauma* 2013.

4 Rajka T, Heyerdahl F, Hovda KE, *et al.* Acute child poisonings in Oslo: a 2-year prospective study. *Acta Paediatr* 2007;**96**:1355–9. doi:10.1111/j.1651-2227.2007.00420.x

5 De Presgrave RF, Camacho LAB, Boas MHSV. A profile of unintentional poisoning caused by household cleaning products, disinfectants and pesticides. *Cad Saude Publica* Published Online First: 2008. doi:10.1590/S0102-311X2008001200019

6 Dayasiri MBKC, Jayamanne SF, Jayasinghe CY. Risk Factors for Acute Unintentional Poisoning among Children Aged 1–5 Years in the Rural Community of Sri Lanka. *Int J*



Confidential: For Review Only

*Pediatr* Published Online First: 2017. doi:10.1155/2017/4375987

7 Schmertmann M, Williamson A, Black D, *et al.* Risk factors for unintentional poisoning in children aged 1-3 years in NSW Australia: A case-control study. *BMC Pediatr* Published Online First: 2013. doi:10.1186/1471-2431-13-88

8 Schmertmann M, Williamson A, Black D. Unintentional poisoning in young children: Does developmental stage predict the type of substance accessed and ingested? *Child Care Health Dev* 2014;**40**:50–9. doi:10.1111/j.1365-2214.2012.01424.x

9 Schwebel DC, Evans WD, Hoeffler SE, *et al.* Unintentional child poisoning risk: A review of causal factors and prevention studies. Child. Heal. Care. 2017. doi:10.1080/02739615.2015.1124775

10 Anwar S, Akmn R, A HSK, *et al.* Clinical Profile of Kerosene Poisoning in a Tertiary Level Hospital in Bangladesh. 2014;**38**:11–4.

11 Seif E, Gomaa R, Eisa M. A retrospective study of acute poisoning in children under 5 years old admitted to Alexandria Poison Center in Egypt. *World J Prev Med* 2016.

12 Wickramasinghe K, Steele P, Dawson A, *et al.* Cost to government health-care services of treating acute self-poisonings in a rural district in Sri Lanka. *Bull World Health Organ* 2009;**87**:180–5. doi:10.2471/BLT.08.051920

13 *Bangladesh Bureau of Statistics (BBS) Statistics and Informatics Division (SID) Ministry of Planning Government of the People's Republic of Bangladesh*. 2013. www.bbs.gov.bd (accessed 13 Feb 2021).

14 BBS. Population & Housing Census-2011. *Bangladesh Bur Stat* 2014.

15 Taylor P, Rahman F, Ali Y, *et al.* Injury Control and Safety Promotion Epidemiology of injury : Results from injury registration at a district level hospital in Bangladesh – implications for prevention in low-income countries Epidemiology of injury : Results from injury registration at a d. 2010;:37–41. doi:10.1076/icsp.8.1.29.3367

16 Rashid MM, Hasan MA, Chowdhury FR. Childhood acute poisoning in a tertiary medical college hospital of Bangladesh. *Mymensingh Med J* 2007;**16**.

Confidential: For Review Only

17 Ahmed F, Rahman MM, Tawfiq M. Clinical profile and outcomes of childhood poisoning. *Bangabandhu Sheikh Mujib Med Univ J* 2017;**10**:264. doi:10.3329/bsmmuj.v10i4.34868

18 He S, Lunnen JC, Puvanachandra P, *et al.* Global Childhood Unintentional Injury Study: Multisite Surveillance Data. *Am J Public Health* 2014;**104**:e79–84. doi:10.2105/AJPH.2013.301607

19 Hyder AA, Wali S, Fishman S, *et al.* The burden of unintentional injuries among the under-five population in South Asia. *Acta Paediatr* 2008;**97**:267–75. doi:10.1111/j.1651-2227.2008.00670.x

20 Ahmed B, Fatmi Z, Siddiqui AR, *et al.* Predictors of unintentional poisoning among children under 5 years of age in karachi: A matched case-control study. *Inj Prev* 2011;**17**:27–33. doi:10.1136/ip.2010.027524

21 Harris PA, Taylor R, Thielke R, *et al.* Research electronic data capture (REDCap)-A metadata-driven methodology and workflow process for providing translational research informatics support. *J Biomed Inform* 2009;**42**:377–81. doi:10.1016/j.jbi.2008.08.010

22 Kohli U, Kuttiat VS, Lodha R, *et al.* Profile of childhood poisoning at a tertiary care centre in north India. *Indian J Pediatr* Published Online First: 2008. doi:10.1007/s12098-008-0105-7

23 Childhood Accidental Poisoning in Tafila. *Child Accid Poisoning Tafila* 2007.

24 Pawłowicz U, Wasilewska A, Olańnski W, *et al.* Epidemiological study of acute poisoning in children: A 5-year retrospective study in the Paediatric University Hospital in Białystok, Poland. *Emerg Med J* Published Online First: 2013. doi:10.1136/emermed-2012-201376

25 Diesel, kerosene prices 23pc up | The Daily Star. https://www.thedailystar.net/news/bangladesh/news/diesel-kerosene-prices-23pc-2221876 (accessed 18 Mar 2022).

26 Yaqoob M, Yar M, Farooq M, *et al.* Acute Poisoning in Children : Etiological Agents , Risk Factors and Outcomes. 2007;**1**:42–4.

Confidential: For Review Only

27 Alazab RM, Elmougy MT, Fayad RA, *et al.* Risk factors of acute poisoning among children: A study at a poisoning unit of a university hospital in Egypt. *South East Asia J Public Heal* 2013;**2**:41–7. doi:10.3329/seajph.v2i2.15943

28 Abbas SK, Tikmani SS, Siddiqui NT. Accidental poisoning in children. *J Pak Med Assoc* 2012;**62**:331–4.

29 Fatmi Z, Kazi A, Hadden WC, *et al.* Incidence and pattern of unintentional injuries and resulting disability among children under 5 years of age: Results of the National Health Survey of Pakistan. *Paediatr Perinat Epidemiol* 2009;**23**:229–38. doi:10.1111/j.1365-3016.2009.01024.x

30 Reimers A, Laflamme L. Neighbourhood social and socio-economic composition and injury risks. *Acta Paediatr Int J Paediatr* 2005;**94**:1488–94. doi:10.1080/08035250510038929

31 Tarvadi PV, Bakkannavar SM, Palimar V, *et al.* Gender Differences in Childhood Poisoning. *J Heal Allied Sci NU* 2013;**03**:069–72. doi:10.1055/s-0040-1703657

32 Jan MA, Siddiqui TS, Ahmed N, *et al.* Mushroom poisoning in children: clinical presentation and outcome. *J Ayub Med Coll Abbottabad* 2008;**20**:99–101.

33 Gauvin F, Bailey B, Bratton SL. Hospitalizations for pediatric intoxication in Washington State, 1987-1997. *Arch Pediatr Adolesc Med* 2001;**155**:1105–10. doi:10.1001/archpedi.155.10.1105

34 Reith DM, Pitt WR, Hockey R. Childhood poisoning in Queensland: An analysis of presentation and admission rates. *J Paediatr Child Health* 2001;**37**:446–50. doi:10.1046/j.1440-1754.2001.00666.x

35 Injuries in the European Union: Summary 2003-2005 - Portail Santé // Grand-Duché de Luxembourg. https://sante.public.lu/fr/publications/i/injuries-eu-stat-2003-2005/index.html (accessed 19 Mar 2022).

36 Dayasiri MBKC, Jayamanne SF, Jayasinghe CY. Patterns and outcome of acute poisoning among children in rural Sri Lanka. *BMC Pediatr* 2018;**18**:1–8. doi:10.1186/s12887-018-1246-0

Confidential: For Review Only

37 Hossain MS. Megacity-centric mass mobility during Eid holidays: a unique concern for infectious disease transmission in Bangladesh. *Trop Med Health* 2022;**50**:4–6. doi:10.1186/s41182-022-00417-4

Confidential: For Review Only

**Table 1: Socio-demographic characteristics of parents and hospitalized children**

| Characteristics | N (%) | CI (95%) |
|---|---|---|
| ***Characteristics of children*** | | |
| **Child age group (years) (n=223)** | | |
| Median age in years (IQR = 75th percentile-25th percentile) | 3 (2.1 = 4-1.9) | |
| **Sex (n=223)** | | |
| Male | 136 (61.0) | (0.54, 0.67) |
| Female | 87 (39.0) | (0.33, 0.45) |
| **Highly active/prompt in nature (n=222)** | | |
| Yes | 146 (65.5) | (0.59, 0.71) |
| No | 76 (34.1) | (0.28, 0.40) |
| ***Characteristics of parents*** | | |
| **Father's age (years) (n=217)** | | |
| Median age | 34 | |
| ≤25 | 9 (4.0) | (0.02, 0.07) |
| 26-30 | 63 (28.3) | (0.23, 0.34) |
| 31-35 | 71 (31.8) | (0.26, 0.38) |
| 36-40 | 44 (19.7) | (0.15, 0.25) |
| >40 | 36 (16.1) | (0.12, 0.21) |
| **Mother's age (years) (n=219)** | | |
| Median age | 28 | |
| ≤25 | 86 (38.6) | (0.32, 0.45) |
| 26-30 | 71 (31.8) | (0.26, 0.38) |
| 31-35 | 53 (23.8) | (0.19, 0.30) |
| 36-40 | 9 (4.0) | (0.02, 0.07) |

Confidential: For Review Only

| | | |
|---|---|---|
| >40 | 4 (1.8) | (0.00, 0.04) |
| **Father's education (n=220)** | | |
| Illiterate | 24 (10.9) | (0.07, 0.15) |
| Primary | 29 (13.2) | (0.09, 0.18) |
| Secondary | 82 (36.3) | (0.31, 0.43) |
| Higher secondary | 54 (24.6) | (0.19, 0.30) |
| Graduation | 31 (14.1) | (0.09, 0.19) |
| **Mother's education (n=222)** | | |
| Illiterate | 28 (12.6) | (0.08, 0.17) |
| Primary | 45 (20.3) | (0.15, 0.26) |
| Secondary | 96 (43.2) | (0.37, 0.49) |
| Higher secondary | 32 (14.4) | (0,10, 0.19) |
| Graduation | 21 (9.5) | (0.06, 0.14) |
| **Father's profession (n=221)** | | |
| Service | 91 (41.2) | (0.34, 0.47) |
| Business | 68 (30.8) | (0.25, 0.37) |
| Farming | 12 (5.4) | (0.03, 0.09) |
| Day labor | 15 (6.8) | (0.04, 0.11) |
| Others | 35 (15.8) | (0.11, 0.21) |
| **Mother's profession (n=222)** | | |
| Housewife | 191 (86.0) | (0.80, .0.89) |
| Service | 15 (6.8) | (0.04, 0.11) |
| Business | 2 (0.9) | (0.00, 0.03) |
| Farming | 2 (0.9) | (0.00, 0.03) |
| Day labor | 0 (0) | (0.00, 0.01) |
| Others | 12 (5.4) | (0.03, 0.09) |
| **Monthly family income (USD) (n=223)** | | |
| <180 | 75 (33.6) | (0.28, 0.40) |
| 180-300 | 103 (46.2) | (0.39, 0.53) |
| 301-590 | 38 (17.0) | (0.13, 0.22) |
| >590 | 7 (3.1) | (0.01, 0.06) |



Confidential: For Review Only

| | | |
|---|---|---|
| **Employment status of parents (n=222)** | | |
| Only the father works outside | 186 (83.8) | (0.78, 0.88) |
| Both parents work outside | 32 (14.4) | (0.10, 0.19) |
| Both parents stay home | 1 (0.45) | (0.00, 0.02) |
| Other | 3 (1.4) | (0.00, 0.04) |
| **Residence (n=223)** | | |
| Metropolitan | 97 (43.5) | (0.37, 0.50) |
| Suburban | 83 (37.2) | (0.31, 0.44) |
| Village | 43 (19.3) | (0.15, 0.25) |
| **Type of family (n=223)** | | |
| Nuclear | 145 (65.0) | (0.58, 0.71) |
| Large | 78 (35.0) | (0.29, 0.41) |

**Table 2: Circumstantial context for accidental poisoning**

| Characteristics | N (%) | CI (95%) |
|---|---|---|
| ***Household factors*** | | |
| **Type of household (n=223)** | | |
| Apartment building | 127 (57.0) | (0.50, 0.63) |
| House made of Tin | 89 (39.9) | (0.34, 0.46) |
| House made of mud/wood/thatch | 6 (2.7) | (0.01, 0.06) |
| Slum | 1 (0.4) | (0.00, 0.02) |
| **Where the incident took place- (n=223)** | | |
| Parents home | 183 (82.1) | (0.76, 0.86) |
| Grandparents' home | 20 (9.0) | (0.05, 0.13) |
| School | 1 (4.0) | (0.00, 0.02) |
| Other | 19 (8.5) | (0.05, 0.13) |
| **In which part of the house the incident took place- (n=223)** | | |

Confidential: For Review Only

| | | |
|---|---|---|
| Bedroom | 114 (51.1) | (0.44, 0.58) |
| Drawing/Dining Room | 21 (9.4) | (0.06, 0.14) |
| Kitchen | 33 (14.8) | (0.10, 0.20) |
| Storeroom | 13 (5.8) | (0.03, 0.09) |
| Yard | 12 (5.4) | (0.03, 0.09) |
| Other | 30 (13.5) | (0.09, 0.18) |
| **Time when the incident took place-** | | |
| Morning | 65 (29.1) | (0.23, 0.35) |
| Noon | 67 (30.0) | (0.24, 0.36) |
| Afternoon | 37 (16.6) | (0.12, 0.22) |
| Night | 54 (24.2) | (0.19, 0.30) |
| **Parents' presence during the incident- (n=223)** | | |
| None | 60 (26.9) | (0.21, 0.33) |
| Only one | 123 (55.2) | (0.48, 0.61) |
| Both | 40 (17.9) | (0.13, 0.23) |
| **Time taken to bring the child to medical attention- (n=223)** | | |
| Less than 2 hours | 150 (67.3) | (0.61, 0.73) |
| 2-4 hours | 54 (24.2) | (0.19, 0.30) |
| 4-6 hours | 15 (6.7) | (0.04, 0.11) |
| More than 6 hours | 4 (1.8) | (0.00, 0.04) |
| ***<u>Safety issues</u>*** | | |
| **The poison was in its original container- (n=223)** | | |
| No | 150 (67.3) | (0.61, 0.73) |
| Yes | 73 (32.7) | (0.27, 0.39) |
| **Chemical stored in soft-drinks' bottles- (n=223)** | | |
| No | 153 (68.6) | (0.62, 0.74) |
| Yes | 70 (31.4) | (0.25, 0.38) |
| **Chemical/medicine stored in an unsafe place at home- (n=223)** | | |

Confidential: For Review Only

| | | |
|---|---|---|
| No | 64 (28.7) | (0.23, 0.35) |
| Yes | 159 (71.3) | (0.65, 0.77) |
| **Event of poisoning happened earlier with the children or with siblings- (n=223)** | | |
| No | 220 (98.7) | (0.96, 0.99) |
| Yes | 3 (1.3) | (0.00, 0.04) |

**Table 3: Cross-tabulation between the type of poisoning and the place in home the incident took place in**

| **Type of poisoning** | **Place in home where incidents took place** | | | | | | |
|---|---|---|---|---|---|---|---|
| | Bedroom | Drawing /Dining room | Kitchen | Store room | Yard | Other | **Total** |
| Kerosene | 28 (38.4%) | 28 (38.4%) | 16 (21.9%) | 4 (5.5%) | 7 (9.6%) | 10 (13.7%) | 73 (100%) |
| Insecticide/ Pesticide | 28 (47.5%) | 6 (10.2%) | 11 (18.6%) | 7 (11.9%) | 3 (5.1%) | 4 (6.8%) | 59 (100%) |
| Medicines | 35 (89.7%) | 2 (5.1%) | 0 (0.0%) | 0 (0.0%) | 0 (0.0%) | 2 (5.1%) | 39 (100%) |
| Household chemicals (bleach/toiletries/phenyl etc.) | 12 (46.2%) | 4 (15.4%) | 3 (11.5%) | 1 (3.8%) | 0 (0.0%) | 6 (23.1%) | 26 (100%) |
| Other | 11 (42.3%) | 1 (3.8%) | 3 (11.5%) | 1 (3.8%) | 2 (7.7%) | 8 (30.8%) | 26 (100%) |
| **Total** | 114 (51.1%) | 21 (9.4%) | 33 (14.8%) | 13 (5.8%) | 12 (5.4%) | 30 (13.5%) | **223 (100%)** |
| $X^2 = 50.48$; $df = 20$; $p = 0.0002$; Fisher's Exact test p = 0.00099 | | | | | | | |

Confidential: For Review Only

391 ## Supplementary files

392 Suppl. Figure 1: The duration of hospital stays

393 The STROBE list of the cross-sectional study

Confidential: For Review Only

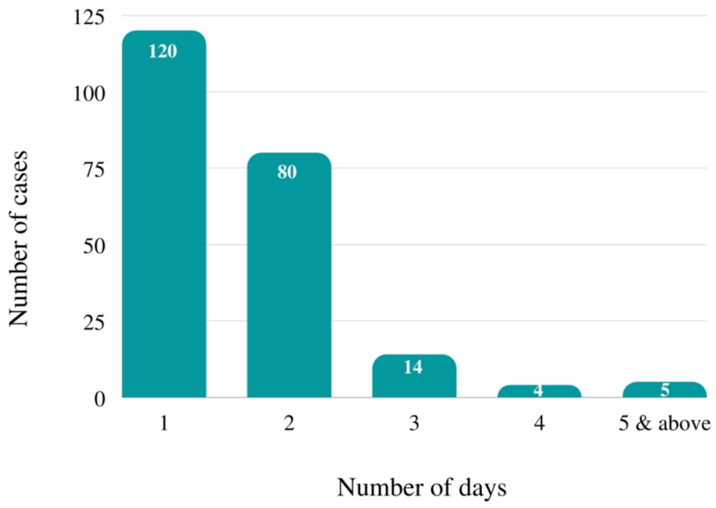


413x269mm (118 x 118 DPI)

**BMJ Paediatrics Open**

# Accidental poisoning in children—a single centre case series study in Bangladesh

| | |
|---|---|
| Journal: | *BMJ Paediatrics Open* |
| Manuscript ID | bmjpo-2022-001541.R2 |
| Article Type: | Original research |
| Date Submitted by the Author: | 21-Jun-2022 |
| Complete List of Authors: | Ahmed, Ahsan; Biomedical Research Foundation; Dhaka Medical College<br>Hasanul Banna, Md.; Biomedical Research Foundation<br>Shojon, Mohammad; Dhaka Medical College<br>Mahdi Hasan, Md.; Biomedical Research Foundation<br>Raheem, Enayetur; Biomedical Research Foundation<br>Hossain, Mohammad; Biomedical Research Foundation |
| Keywords: | Epidemiology |
| | |

SCHOLARONE™
Manuscripts

BMJ

*I, the Submitting Author has the right to grant and does grant on behalf of all authors of the Work (as defined in the below author licence), an exclusive licence and/or a non-exclusive licence for contributions from authors who are: i) UK Crown employees; ii) where BMJ has agreed a CC-BY licence shall apply, and/or iii) in accordance with the terms applicable for US Federal Government officers or employees acting as part of their official duties; on a worldwide, perpetual, irrevocable, royalty-free basis to BMJ Publishing Group Ltd ("BMJ") its licensees and where the relevant Journal is co-owned by BMJ to the co-owners of the Journal, to publish the Work in this journal and any other BMJ products and to exploit all rights, as set out in our licence.*

*The Submitting Author accepts and understands that any supply made under these terms is made by BMJ to the Submitting Author unless you are acting as an employee on behalf of your employer or a postgraduate student of an affiliated institution which is paying any applicable article publishing charge ("APC") for Open Access articles. Where the Submitting Author wishes to make the Work available on an Open Access basis (and intends to pay the relevant APC), the terms of reuse of such Open Access shall be governed by a Creative Commons licence – details of these licences and which Creative Commons licence will apply to this Work are set out in our licence referred to above.*

*Other than as permitted in any relevant BMJ Author's Self Archiving Policies, I confirm this Work has not been accepted for publication elsewhere, is not being considered for publication elsewhere and does not duplicate material already published. I confirm all authors consent to publication of this Work and authorise the granting of this licence.*

Confidential: For Review Only

# Accidental poisoning in children—a single centre case series study in Bangladesh

Ahsan Ahmed[1, 2], Md. Hasanul Banna Siam[1], Mohammad Shojon[2], Md. Mahdi Hasan[1], Enayetur Raheem[1] and Mohammad Sorowar Hossain[1,3]

1 Department of Emerging and Neglected Diseases, Biomedical Research Foundation, Dhaka, Bangladesh

[2]Dhaka Medical College, Dhaka, Bangladesh

[3]School of Environment and Life Sciences, Independent University, Bangladesh, Dhaka, Bangladesh

*** Correspondence:**

Mohammad Sorowar Hossain

Email: sorowar.hossain@brfbd.org

**Keywords:** Injury, Prevention, Epidemiology, Poisoning, Children, Dhaka, Bangladesh

Confidential: For Review Only

# Abstract

## Background

Accidental poisoning is a leading cause of unintentional injuries among children in lower- and middle-income (LMICs) countries. The overall aspect of this unintentional poisoning is poorly understood in Bangladesh. The objectives of this study were 1) to explore the socio-demographic factors and circumstantial context of accidental poisoning and 2) the prevalence of the type of substances causing it.

## Methods

A descriptive case series study was conducted from April, 2019 to February, 2020 at a tertiary level hospital of the capital city Dhaka in Bangladesh. Children under 10 years of age admitted to the hospital with accidental poisoning were enrolled in this study. Parents of hospitalized children were interviewed face-to-face using a structured questionnaire. Descriptive statistics were used for data analysis.

## Results

A total of 223 children were recruited in this study. Children between 2 to 5 years (60%), male (61%), and children with agile (65.5%) were among the prevalent victims. The majority of cases occurred (65%) in a nuclear family setting. Most mothers (85%) of these children were non-working and most incidents took place in parents' homes (~82%). Nearly 70% of the poisoning incidents took place in the presence of parents and over half of these occurred in the bedroom. Kerosene was the prevalent cause (33%) of accidental poisoning while insecticide/pesticide ranked second (26.5%) followed by medicines (17%) and household chemicals (12). In one-third (31.4%) cases, poisoning chemicals were stored in soft-drinks bottles while two-thirds (67.3%) were kept in containers other than original ones. Although over 80 parents somewhat knew that chemicals could be harmful to the children if ingested, most of them did not take the safety measures.


Confidential: For Review Only

**Conclusion**

In this present study we found that preschool-aged children were more victims of accidental poisoning mostly by ingesting kerosene and a majority of the incidents took place in the bedroom while parents were present at home. Our study findings would serve as a baseline for designing future intervention studies and policies.

## What is already known on this subject

- Poisoning is a leading cause of morbidity and mortality as an unintentional injury all over the world which is seen preventable in many settings by taking region-specific measures.
- High income countries (HICs) have achieved significant reduction of poisoning incidents by taking definite measures but low- and middle-income countries lag behind due to gap in knowledge and insights to prevent it.

## What this study adds

- As a lower middle-income country, in-house settings of poisoning cases have been explored in this study.
- Kerosene and insecticides/pesticides were the most frequent poisoning substances. Poisonous chemicals often stored in soft drink bottles/containers.

## How this study might affect research, practice or policy

- This study may open up the scope of extensive research into in-house settings regarding accidental poisoning.
- This study may play a baseline role to adopt significant changes in raising awareness about proper disposal of soft drinks bottles and confronting soft-drink companies to play role for that purpose.
- The policymakers should rethink controlling the use and bringing advanced regulatory measures regarding practice of kerosene, insecticides, pesticides and other harmful chemicals use.

Confidential: For Review Only

# Introduction

Globally, poisoning is one of the leading causes of all unintentional injuries in children [1]. Poisonous substances can cause significant morbidity or mortality when ingested, inhaled, injected, or absorbed through the skin in a high concentration. Acute toxicity is linked with long-term neuropsychiatric health consequences [2–4]. According to the World Health Organization, nearly 200,000 people die each year due to accidental poisoning. Around 80% of these deaths occur in low- and middle-income countries (LMICs). Accidental poisoning accounts for 10% of the total burden of unintentional injuries among children in lower- and middle-income countries (LMICs) [1,5,6]. These incidents are associated with a number of factors that have been identified in different settings [7–9]. Notably, about 15% of unintentional poisoning-related deaths occur below the age of 5 years and many cases go unreported or under-reported, or misreported [10].

The causative factors and outcome of unintentional poisoning cases are influenced by various socio-demographic and economic factors and also by the availability and the quality of the medical facilities in any given region [2,9,11]. Accidental poisoning has also an economic impact on the family to get medical treatment for the victim as most of the cases are from LMICs. In a study conducted in Sri Lanka, on an average 31.83 USD was spent per patient for treatment purposes, with an addition of 14.03 USD per patient in transport [12].

Bangladesh is a low-middle income nation with over 30 million children under the age of 10 [13,14]. A nationwide survey in 1997-1998 estimated an incidence of 11 poisoning cases per 100,000 per year in Bangladesh [15]. Unlike high-income countries, there is no awareness or practice of using child-proof containers for poisonous substances in Bangladesh. Government directives also have not properly addressed the issue of using child-protective containers for chemical agents. Even though accidental poisoning is one of the leading causes of unintentional injury among children in LMICs, it has received little attention in Bangladesh. There is no comprehensive epidemiological study focusing household factors for accidental poisoning among children in Bangladesh [10,16–18]. All the prior studies here had a small sample size and primarily focused on the prevalence of chemicals that caused accidental poisoning and on outcomes. Thus, we aimed to investigate the current prevalence of the type of chemical agents causing accidental



Confidential: For Review Only

poisoning among children and related socio-demographic and circumstantial context in children admitted in one of the largest tertiary level hospitals in Bangladesh.

## Methods

***Study site and sample size***

This descriptive case series study was conducted from 20 April, 2019 to 21 February, 2020 at Dhaka Medical College Hospital, a tertiary level hospital situated at the capital city Dhaka in Bangladesh. Notably, data collection was completed before the declaration of the COVID-19 pandemic in Bangladesh (first case was reported on 8 March 2020). The hospital has one of the highest capacities to accommodate patients and the highest level of facilities for all the economic classes of the country. The admission capacity of the pediatric medicine department is approximately 30 children per day but around twice as many patients are admitted usually. Moreover, the hospital mostly serves middle to lower-income class people.This study enrolled children under 10 years of age who wereadmitted via the emergency department with a diagnosis of poisoning (unintentional) through convenience sampling approach. Children above 10 years of age and cases with food poisoning, adverse drug reactions, snake bite, animal venom, or insect bite were excluded.

***Definition of poisoning substances***

A poisonous substance was defined as any agent that had the capacity or potential to produce toxic effects or morbidity or mortality to the child while ingested, inhaled, injected, or absorbed through the skin in quantities enough to cause physiological and neuropsychological effects. Thus, compounds such as pharmaceuticals, petroleum products, household chemicals, insecticides, and pesticides were considered poisonous in this study.

***Questionnaire and data collection***

The study questionnaire (added in supplementary materials) was developed based on existing literature and expert opinion (including a pediatrician and public health researcher) to understand the epidemiology and possible factors related to childhood accidental poisoning in Bangladesh [19,20]. In the questionnaire, basic demographic information included: age of the child, parents' age and their marital status, education, profession, monthly family income, place of living

Confidential: For Review Only

(urban/semi-urban/rural). One question was asked about the child's agility (yes/no). Questions related to poisoning included: type of poisonous substance ingested/inhaled, time and exact place of the incidence, parents' presence at that time, time taken to seek medical care, type of container, and how chemicals and medicines were stored in the house. Parents/guardians were asked: whether they knew the substance taken by the child was harmful (yes/no), whether they alerted the child about the harmful sides of stored chemicals/medicines/kerosene (yes/no). The draft questionnaire was piloted on 5 parents and revised accordingly.

Face-to-face interviews with children's attendants were conducted using a structured questionnaire in the native language (Bangla). Two of the authors (medical student) and an intern physician took part in the data collection procedure. No children were interviewed; instead, information about accidental poisoning incidents was provided by their parents or guardians.

***Statistical analysis***

Data were stored and secured in Research Electronic Data Capture (REDCap) platform hosted at BRF [21]. Descriptive statistical analysis was performed. When applicable, descriptive data were expressed as percentages and means, along with standard deviation. Pearson's chi-square test was performed to determine the association between categorical data (time of incident took place and presence of parents at home; type of poisoning and place in home where the incident took place) and a p-value smaller than 0.05 was considered statistically significant.

***Ethical consideration***

The study protocol was approved by the institutional review board of Biomedical Research Foundation, Bangladesh (Ref. no: BRF/ERB/2019/004). Each subject provided verbal and/or written informed consent at their discretion. The parents were approached first and informed about the study's nature and objective. Following consent (oral and/or written), participants were registered for an interview. Some respondents were unable to sign their names; in these instances, the questionnaire was marked as a verbal agreement. In the context of Bangladesh, patients or patients' attendants usually do not refuse to participate in such interview sessions in public hospitals. No patients' attendants declined to participate in this study. For data collection, approval was taken from the head of the pediatric medicine department, Dhaka Medical College Hospital.

Confidential: For Review Only

Parents were assured with good faith that their shared information would be confidential under doctor-patient relationship oath so that they could talk freely about the incident.

***Patient participation statement***

Patients were involved in the design stage, questionnaire development and conduct of the research. The questionnaire was trialled with 5 patients and was modified as per observation and respondents' experience. Over the period of data collection, it was ensured that the patients participate in the study when they are stable after a hectic poisoning accident. Moreover, patients' welfare in the crowded hospital was a priority. We intend to disseminate the study results in mass public to aware them as well as to policy levels to mitigate the preventable poisoning accidents.

## Results

A total of 223 children with accidental poisoning were enrolled in the study. In 60% of cases, children were aged between 2 and 5 years and male children (61%) were the major victim. Around two-thirds (65.5%) of the children were highly active in behavior or prompt in nature (means they were up and doing and more active than peers according to the parents) (**Table 1**). Nearly 43% of the mothers had more than 10 years of schooling while it was 36.8% for fathers. More than 85% of mothers were stay-at-home mothers (**Table 1**). The majority of incidents occurred in a nuclear family setting (65%). In most families, fathers were the bread-winner (83%); and the monthly incomes of a major portion of the families (79.8%) were below USD 300 (**Table 1**).

**Table 2** reports the circumstantial context of poisoning incidence. More than two-thirds of the incidents (82.1%) took place in parents' homes and 57% of those were in apartment buildings. Within the house, half of the cases (51.1%) took place in bedrooms, while 14.8% occurred in the kitchen. About 30% of incidents occurred before noon while 24.2% of incidents took place at night and 16.6% in the afternoon. Over two-thirds of incidents (70.9%) occurred in the presence of at least one parent at home while nearly 30% of incidents took place when neither of the parents were at home. Interestingly, incidents during the morning were linked to both parents being outside, while nighttime incidents were linked to both parents being at home ($p<0.005$).

Ingestion of kerosene was caused in nearly one-third (32.7%) of hospitalized cases, with insecticides/pesticides being the second most common cause (26.5%). Poisoning from ingestion of medicines was found to occur in 17.5% of cases, whereas poisoning due to household chemicals



Confidential: For Review Only

such as bleach/toiletries/phenyl occurred in 11.7% of cases. Other substances were responsible for 11.7% of the cases. Type of poisoning was found to associated with the place in home the incident took place (p<0.005) (**table 3**).

As for the storage of chemical/medicine, about 71.3% of the respondents reported that the chemical/medicine substances were kept in places within the child's reach. In one-third of the cases (31.4%), chemicals such as kerosene were stored in bottles of soft drinks.

Almost all incidents (98.7%) took place for the first time in the family. About 20% of parents were unaware whether ingested substances were harmful. Most parents (78.4%) did not take protective measures for storing chemicals at home. Two-thirds of the children (67.3%) were taken to medical attention within 2 hours, while 24.2% were within 2-4 hours and 8.5% in more than 4 hours. More than half the children (53.8%) stayed at the hospital for a day, while 39.5% of them stayed for 2 days (**Suppl. Figure 1**). The mean hospital stay was 1.8 days (SD: 2.42).

## Discussion

To the best of our knowledge, this is the first comprehensive study from Bangladesh to understand the prevalence and characteristics of the cases of unintentional poisoning in children. We found that kerosene was the most prevalent poisoning agent and a majority of the incidents took place in the bedrooms while parents were present at home.

In South Asian countries, kerosene remains the most prevalent cause of accidental poisoning among children. Our study finding is also consistent with previous studies from this region [2,20,22,23]. In contrast, household chemicals and medicine are the main causes of children's accidental poisoning in HICs [2–4,11,24]. Bangladesh consumes 100,000 tons of kerosene a year [25]. This is mainly used for cooking and lightening in shanties in the metropolitans, peri-urban and rural areas where there is no or limited service for natural gas. Most hospitalized children in the present study came from underdeveloped (shanties and peri-urban) areas. In nearly all kerosene poisoning cases, liquid hydrocarbon was stored in soft drink bottles (plastic). As a consequence, children are deceived to think of kerosene as soft drinks. A similar practice is also reported from some other South Asian countries [20,26]. Single-use plastic bottles/containers, especially soft drinks bottles are repeatedly used in LMICs for storing food and liquids. Given the popularity of soft drinks particularly Coca Cola, Pepsi, Sprite and other local ones, storing kerosene or liquid



https://mc.manuscriptcentral.com/bmjpo

Confidential: For Review Only

chemicals in these empty soft drink bottles is very common in day-to-day life here. Therefore, popular soft drinks companies should also contribute to raise community-level awareness to prevent accidental poisoning in children, particularly in LMICs.

Despite 85% of mothers being housewives (stay-at-home), over 70% of accidental incidents occurred when parents were at home. Similar to prior studies inLMICs, most mothers were younger [27–30], and nearly all incidents occurred for the first time in the family. Similar to other studies, preschool-aged male children were more prone to accidental poisoning [31–35]. Lack of experience in parenting and not taking practical measures might contribute to these incidents. Even though most parents were aware of the potential accident, they were not able to translate their knowledge into practice. This psychological paradox in this region warrants further study.

The home environmental factors might have served as barriers to proper safety practice since bedrooms were the commonest place where half of the incidents took place in our study. However, prior studies reported kitchens and yards were the prevalent locations for accidental poisoning [36]. In our study, most poisoning cases were from disadvantaged families (lower economic stratum) who were mostly living in small apartment houses with only one or two small rooms. Due to lack of enough space, all the daily chores and commodities are kept and surrounded in a crowded manner in these small houses. Moreover, Bangladeshi families hardly practice store room keeping and usually store substances near to hand. Because of the exploratory nature of children, poisonous substances easily find their way into the hands of children. Effective parental (mother-centric) awareness and the use of child-protective containers could be viable solutions for preventing unintentional childhood poisoning.

## Limitations

A large sample and in-house context exploration are the strengths of this study. However, most childhood poisoning cases were from metropolitan or nearby areas. Therefore, our study did not portray the scenario of rural areas where nearly 70% of the Bangladeshi population live . Our findings also may not be generalizable to other urban areas of the country as Dhaka city is the most crowded megacity in the world [37]. However, our study findings could be useful for similarly crowded megacities inLMICs. In Bangladesh, patients with a lower economic background generally seek healthcare services in public hospitals and thus our findings are not representative of all economic classes. Moreover, since we used a convenience sampling approach for enrolling





Confidential: For Review Only

patients, this may introduce a bias in the outcome and therefore our findings should be interpreted with caution.

## Conclusion

In this present study, we found that kerosene was the most prevalent causative agent of poisoning among children and a majority of the incidents took place in the bedroom while parents were present at home. Our study findings would serve as a baseline for designing future intervention studies (such as mother-centric prevention) and policies to make soft-drink companies socially responsible and could also enforce the issue of child protective containers.

## Declarations

### Funding

This research received no specific grant from any funding agency in the public, commercial or not-for-profit sectors.

### Competing interests

The authors declare that there is no conflict of interest regarding the publication of this article.

### Author contributions

MSH conceived the idea. MSH and AA designed and developed the questionnaire. ER designed the study. AA led the data collection stage along with MS. ER along with MMH analyzed data. AA, MSH, MHBS, MS, ER, MMH contributed in the result preparation. AA prepared the draft and MHBS and MSH reviewed and revised it.

### Acknowledgement

The authors would like to impart their immense gratitude to Dr. Golam Moktadir for contributing and all the doctors and nurses of the pediatric medicine department of Dhaka Medical College for being supportive at the data collection stage. The authors are especially grateful to Prof. Sayeeda Anwar, the then head of the pediatric medicine department of Dhaka Medical College Hospital



Confidential: For Review Only

and Prof. Zohora Jameela Khan, the head of the pediatric hematology and oncology department of Dhaka Medical College Hospital for providing required assistance needed at the study site.

**Patient consent form**

It is not applicable for the study as no information of any identifiable individual is disclosed.

**Authors' information**

Affiliations

*Biomedical Research Foundation, Dhaka-1230, Bangladesh*

Ahsan Ahmed, Md. Hasanul Banna Siam, Md. Mahdi Hasan, Enayetur Raheem

*Dhaka Medical College, Dhaka, Bangladesh*

Ahsan Ahmed, Mohammad Sojon.

## References

1 Chandran A, Hyder AA, Peek-Asa C. The global burden of unintentional injuries and an agenda for progress. *Epidemiol Rev* 2010;**32**:110–20. doi:10.1093/epirev/mxq009

2 Özdemir R, Bayrakci B, Tekşam Ö, *et al.* Thirty-three-year experience on childhood poisoning. *Turk J Pediatr* 2012.

3 Haghighat M, Moravej H, Moatamedi M. Epidemiology of Pediatric Acute Poisoning in Southern Iran: A Hospital-Based Study. *Bull Emerg trauma* 2013.

4 Rajka T, Heyerdahl F, Hovda KE, *et al.* Acute child poisonings in Oslo: a 2-year prospective study. *Acta Paediatr* 2007;**96**:1355–9. doi:10.1111/j.1651-2227.2007.00420.x

5 De Presgrave RF, Camacho LAB, Boas MHSV. A profile of unintentional poisoning caused by household cleaning products, disinfectants and pesticides. *Cad Saude Publica* Published Online First: 2008. doi:10.1590/S0102-311X2008001200019

6 Dayasiri MBKC, Jayamanne SF, Jayasinghe CY. Risk Factors for Acute Unintentional Poisoning among Children Aged 1–5 Years in the Rural Community of Sri Lanka. *Int J*



Confidential: For Review Only

*Pediatr* Published Online First: 2017. doi:10.1155/2017/4375987

7 Schmertmann M, Williamson A, Black D, *et al.* Risk factors for unintentional poisoning in children aged 1-3 years in NSW Australia: A case-control study. *BMC Pediatr* Published Online First: 2013. doi:10.1186/1471-2431-13-88

8 Schmertmann M, Williamson A, Black D. Unintentional poisoning in young children: Does developmental stage predict the type of substance accessed and ingested? *Child Care Health Dev* 2014;**40**:50–9. doi:10.1111/j.1365-2214.2012.01424.x

9 Schwebel DC, Evans WD, Hoeffler SE, *et al.* Unintentional child poisoning risk: A review of causal factors and prevention studies. Child. Heal. Care. 2017. doi:10.1080/02739615.2015.1124775

10 Anwar S, Akmn R, A HSK, *et al.* Clinical Profile of Kerosene Poisoning in a Tertiary Level Hospital in Bangladesh. 2014;**38**:11–4.

11 Seif E, Gomaa R, Eisa M. A retrospective study of acute poisoning in children under 5 years old admitted to Alexandria Poison Center in Egypt. *World J Prev Med* 2016.

12 Wickramasinghe K, Steele P, Dawson A, *et al.* Cost to government health-care services of treating acute self-poisonings in a rural district in Sri Lanka. *Bull World Health Organ* 2009;**87**:180–5. doi:10.2471/BLT.08.051920

13 *Bangladesh Bureau of Statistics (BBS) Statistics and Informatics Division (SID) Ministry of Planning Government of the People's Republic of Bangladesh*. 2013. www.bbs.gov.bd (accessed 13 Feb 2021).

14 BBS. Population & Housing Census-2011. *Bangladesh Bur Stat* 2014.

15 Taylor P, Rahman F, Ali Y, *et al.* Injury Control and Safety Promotion Epidemiology of injury : Results from injury registration at a district level hospital in Bangladesh – implications for prevention in low-income countries Epidemiology of injury *Inj Control and Safety Promotion* 2010;:37–41. doi:10.1076/icsp.8.1.29.3367

16 Rashid MM, Hasan MA, Chowdhury FR. Childhood acute poisoning in a tertiary medical college hospital of Bangladesh. *Mymensingh Med J* 2007;**16**.

Confidential: For Review Only

17 Ahmed F, Rahman MM, Tawfiq M. Clinical profile and outcomes of childhood poisoning. *Bangabandhu Sheikh Mujib Med Univ J* 2017;**10**:264. doi:10.3329/bsmmuj.v10i4.34868

18 He S, Lunnen JC, Puvanachandra P, *et al.* Global Childhood Unintentional Injury Study: Multisite Surveillance Data. *Am J Public Health* 2014;**104**:e79–84. doi:10.2105/AJPH.2013.301607

19 Hyder AA, Wali S, Fishman S, *et al.* The burden of unintentional injuries among the under-five population in South Asia. *Acta Paediatr* 2008;**97**:267–75. doi:10.1111/j.1651-2227.2008.00670.x

20 Ahmed B, Fatmi Z, Siddiqui AR, *et al.* Predictors of unintentional poisoning among children under 5 years of age in karachi: A matched case-control study. *Inj Prev* 2011;**17**:27–33. doi:10.1136/ip.2010.027524

21 Harris PA, Taylor R, Thielke R, *et al.* Research electronic data capture (REDCap)-A metadata-driven methodology and workflow process for providing translational research informatics support. *J Biomed Inform* 2009;**42**:377–81. doi:10.1016/j.jbi.2008.08.010

22 Kohli U, Kuttiat VS, Lodha R, *et al.* Profile of childhood poisoning at a tertiary care centre in north India. *Indian J Pediatr* Published Online First: 2008. doi:10.1007/s12098-008-0105-7

23 Childhood Accidental Poisoning in Tafila. *Child Accid Poisoning Tafila* 2007.

24 Pawłowicz U, Wasilewska A, Olańnski W, *et al.* Epidemiological study of acute poisoning in children: A 5-year retrospective study in the Paediatric University Hospital in Białystok, Poland. *Emerg Med J* Published Online First: 2013. doi:10.1136/emermed-2012-201376

25 Diesel, kerosene prices 23pc up | The Daily Star. https://www.thedailystar.net/news/bangladesh/news/diesel-kerosene-prices-23pc-2221876 (accessed 18 Mar 2022).

26 Yaqoob M, Yar M, Farooq M, *et al.* Acute Poisoning in Children : Etiological Agents , Risk Factors and Outcomes. *Pak J of Med and Health Sci* 2007;**1**:42–4.

Confidential: For Review Only

27 Alazab RM, Elmougy MT, Fayad RA, *et al.* Risk factors of acute poisoning among children: A study at a poisoning unit of a university hospital in Egypt. *South East Asia J Public Heal* 2013;**2**:41–7. doi:10.3329/seajph.v2i2.15943

28 Abbas SK, Tikmani SS, Siddiqui NT. Accidental poisoning in children. *J Pak Med Assoc* 2012;**62**:331–4.

29 Fatmi Z, Kazi A, Hadden WC, *et al.* Incidence and pattern of unintentional injuries and resulting disability among children under 5 years of age: Results of the National Health Survey of Pakistan. *Paediatr Perinat Epidemiol* 2009;**23**:229–38. doi:10.1111/j.1365-3016.2009.01024.x

30 Reimers A, Laflamme L. Neighbourhood social and socio-economic composition and injury risks. *Acta Paediatr Int J Paediatr* 2005;**94**:1488–94. doi:10.1080/08035250510038929

31 Tarvadi PV, Bakkannavar SM, Palimar V, *et al.* Gender Differences in Childhood Poisoning. *J Heal Allied Sci NU* 2013;**03**:069–72. doi:10.1055/s-0040-1703657

32 Jan MA, Siddiqui TS, Ahmed N, *et al.* Mushroom poisoning in children: clinical presentation and outcome. *J Ayub Med Coll Abbottabad* 2008;**20**:99–101.

33 Gauvin F, Bailey B, Bratton SL. Hospitalizations for pediatric intoxication in Washington State, 1987-1997. *Arch Pediatr Adolesc Med* 2001;**155**:1105–10. doi:10.1001/archpedi.155.10.1105

34 Reith DM, Pitt WR, Hockey R. Childhood poisoning in Queensland: An analysis of presentation and admission rates. *J Paediatr Child Health* 2001;**37**:446–50. doi:10.1046/j.1440-1754.2001.00666.x

35 Injuries in the European Union: Summary 2003-2005 - Portail Santé // Grand-Duché de Luxembourg. https://sante.public.lu/fr/publications/i/injuries-eu-stat-2003-2005/index.html (accessed 19 Mar 2022).

36 Dayasiri MBKC, Jayamanne SF, Jayasinghe CY. Patterns and outcome of acute poisoning among children in rural Sri Lanka. *BMC Pediatr* 2018;**18**:1–8. doi:10.1186/s12887-018-1246-0

Confidential: For Review Only

37 Hossain MS. Megacity-centric mass mobility during Eid holidays: a unique concern for infectious disease transmission in Bangladesh. *Trop Med Health* 2022;**50**:4–6. doi:10.1186/s41182-022-00417-4

Confidential: For Review Only

**Table 1: Socio-demographic characteristics of parents and hospitalized children**

| Characteristics | N (%) | CI (95%) |
|---|---|---|
| ***Characteristics of children*** | | |
| **Child age group (years) (n=223)** | | |
| Median age in years (25th percentile-75th percentile) | 3 (1.9-4) | |
| **Sex (n=223)** | | |
| Male | 136 (61.0) | (0.54, 0.67) |
| Female | 87 (39.0) | (0.33, 0.45) |
| **Highly active/prompt in nature (n=222)** | | |
| Yes | 146 (65.5) | (0.59, 0.71) |
| No | 76 (34.1) | (0.28, 0.40) |
| ***Characteristics of parents*** | | |
| **Father's age (years) (n=217)** | | |
| Median age | 34 | |
| ≤25 | 9 (4.0) | (0.02, 0.07) |
| 26-30 | 63 (28.3) | (0.23, 0.34) |
| 31-35 | 71 (31.8) | (0.26, 0.38) |
| 36-40 | 44 (19.7) | (0.15, 0.25) |
| >40 | 36 (16.1) | (0.12, 0.21) |
| **Mother's age (years) (n=219)** | | |
| Median age | 28 | |
| ≤25 | 86 (38.6) | (0.32, 0.45) |
| 26-30 | 71 (31.8) | (0.26, 0.38) |
| 31-35 | 53 (23.8) | (0.19, 0.30) |
| 36-40 | 9 (4.0) | (0.02, 0.07) |
| >40 | 4 (1.8) | (0.00, 0.04) |
| **Father's education (n=220)** | | |
| Illiterate | 24 (10.9) | (0.07, 0.15) |

Confidential: For Review Only

| | | |
|---|---|---|
| Primary | 29 (13.2) | (0.09, 0.18) |
| Secondary | 82 (36.3) | (0.31, 0.43) |
| Higher secondary | 54 (24.6) | (0.19, 0.30) |
| Graduation | 31 (14.1) | (0.09, 0.19) |
| **Mother's education (n=222)** | | |
| Illiterate | 28 (12.6) | (0.08, 0.17) |
| Primary | 45 (20.3) | (0.15, 0.26) |
| Secondary | 96 (43.2) | (0.37, 0.49) |
| Higher secondary | 32 (14.4) | (0,10, 0.19) |
| Graduation | 21 (9.5) | (0.06, 0.14) |
| **Father's profession (n=221)** | | |
| Service | 91 (41.2) | (0.34, 0.47) |
| Business | 68 (30.8) | (0.25, 0.37) |
| Farming | 12 (5.4) | (0.03, 0.09) |
| Day labor | 15 (6.8) | (0.04, 0.11) |
| Others | 35 (15.8) | (0.11, 0.21) |
| **Mother's profession (n=222)** | | |
| Housewife | 191 (86.0) | (0.80, .0.89) |
| Service | 15 (6.8) | (0.04, 0.11) |
| Business | 2 (0.9) | (0.00, 0.03) |
| Farming | 2 (0.9) | (0.00, 0.03) |
| Day labor | 0 (0) | (0.00, 0.01) |
| Others | 12 (5.4) | (0.03, 0.09) |
| **Monthly family income (USD) (n=223)** | | |
| <180 | 75 (33.6) | (0.28, 0.40) |
| 180-300 | 103 (46.2) | (0.39, 0.53) |
| 301-590 | 38 (17.0) | (0.13, 0.22) |
| >590 | 7 (3.1) | (0.01, 0.06) |
| **Employment status of parents (n=222)** | | |
| Only the father works outside | 186 (83.8) | (0.78, 0.88) |
| Both parents work outside | 32 (14.4) | (0.10, 0.19) |

Confidential: For Review Only

| | | |
|---|---|---|
| Both parents stay home | 1 (0.45) | (0.00, 0.02) |
| Other | 3 (1.4) | (0.00, 0.04) |
| **Residence (n=223)** | | |
| Metropolitan | 97 (43.5) | (0.37, 0.50) |
| Suburban | 83 (37.2) | (0.31, 0.44) |
| Village | 43 (19.3) | (0.15, 0.25) |
| **Type of family (n=223)** | | |
| Nuclear | 145 (65.0) | (0.58, 0.71) |
| Large | 78 (35.0) | (0.29, 0.41) |

Confidential: For Review Only

**Table 2: Circumstantial context for accidental poisoning**

| **Characteristics** | **N (%)** | **CI (95%)** |
|---|---|---|
| ***Household factors*** | | |
| **Type of household (n=223)** | | |
| Apartment building | 127 (57.0) | (0.50, 0.63) |
| House made of Tin | 89 (39.9) | (0.34, 0.46) |
| House made of mud/wood/thatch | 6 (2.7) | (0.01, 0.06) |
| Slum | 1 (0.4) | (0.00, 0.02) |
| **Where the incident took place- (n=223)** | | |
| Parents home | 183 (82.1) | (0.76, 0.86) |
| Grandparents' home | 20 (9.0) | (0.05, 0.13) |
| School | 1 (4.0) | (0.00, 0.02) |
| Other | 19 (8.5) | (0.05, 0.13) |
| **In which part of the house the incident took place- (n=223)** | | |
| Bedroom | 114 (51.1) | (0.44, 0.58) |
| Drawing/Dining Room | 21 (9.4) | (0.06, 0.14) |
| Kitchen | 33 (14.8) | (0.10, 0.20) |
| Storeroom | 13 (5.8) | (0.03, 0.09) |
| Yard | 12 (5.4) | (0.03, 0.09) |
| Other | 30 (13.5) | (0.09, 0.18) |
| **Time when the incident took place-** | | |
| Morning | 65 (29.1) | (0.23, 0.35) |
| Noon | 67 (30.0) | (0.24, 0.36) |
| Afternoon | 37 (16.6) | (0.12, 0.22) |
| Night | 54 (24.2) | (0.19, 0.30) |
| **Parents' presence during the incident- (n=223)** | | |
| None | 60 (26.9) | (0.21, 0.33) |
| Only one | 123 (55.2) | (0.48, 0.61) |

Confidential: For Review Only

| | | |
|---|---|---|
| Both | 40 (17.9) | (0.13, 0.23) |
| **Time taken to bring the child to medical attention- (n=223)** | | |
| Less than 2 hours | 150 (67.3) | (0.61, 0.73) |
| 2-4 hours | 54 (24.2) | (0.19, 0.30) |
| 4-6 hours | 15 (6.7) | (0.04, 0.11) |
| More than 6 hours | 4 (1.8) | (0.00, 0.04) |
| ***Safety issues*** | | |
| **The poison was in its original container- (n=223)** | | |
| No | 150 (67.3) | (0.61, 0.73) |
| Yes | 73 (32.7) | (0.27, 0.39) |
| **Chemical stored in soft-drinks' bottles- (n=223)** | | |
| No | 153 (68.6) | (0.62, 0.74) |
| Yes | 70 (31.4) | (0.25, 0.38) |
| **Chemical/medicine stored in an unsafe place at home- (n=223)** | | |
| No | 64 (28.7) | (0.23, 0.35) |
| Yes | 159 (71.3) | (0.65, 0.77) |
| **Event of poisoning happened earlier with the children or with siblings- (n=223)** | | |
| No | 220 (98.7) | (0.96, 0.99) |
| Yes | 3 (1.3) | (0.00, 0.04) |

Confidential: For Review Only

392 **Table 3: Cross-tabulation between the type of poisoning and the place in home the incident**
393 **took place in**

| **Type of poisoning** | **Place in home where incidents took place** | | | | | | |
|---|---|---|---|---|---|---|---|
| | Bedroom | Drawing /Dining room | Kitchen | Store room | Yard | Other | **Total** |
| Kerosene | 28 (38.4%) | 28 (38.4%) | 16 (21.9%) | 4 (5.5%) | 7 (9.6%) | 10 (13.7%) | 73 (100%) |
| Insecticide/ Pesticide | 28 (47.5%) | 6 (10.2%) | 11 (18.6%) | 7 (11.9%) | 3 (5.1%) | 4 (6.8%) | 59 (100%) |
| Medicines | 35 (89.7%) | 2 (5.1%) | 0 (0.0%) | 0 (0.0%) | 0 (0.0%) | 2 (5.1%) | 39 (100%) |
| Household chemicals (bleach/toiletries/phenyl etc.) | 12 (46.2%) | 4 (15.4%) | 3 (11.5%) | 1 (3.8%) | 0 (0.0%) | 6 (23.1%) | 26 (100%) |
| Other | 11 (42.3%) | 1 (3.8%) | 3 (11.5%) | 1 (3.8%) | 2 (7.7%) | 8 (30.8%) | 26 (100%) |
| **Total** | 114 (51.1%) | 21 (9.4%) | 33 (14.8%) | 13 (5.8%) | 12 (5.4%) | 30 (13.5%) | **223 (100%)** |
| $X^2 = 50.48$; $df = 20$; $p = 0.0002$; Fisher's Exact test p = 0.00099 | | | | | | | |

394

395

396 ## Supplementary files

397 Suppl. Figure 1: The duration of hospital stays

398 The STROBE list of the cross-sectional study



Confidential: For Review Only

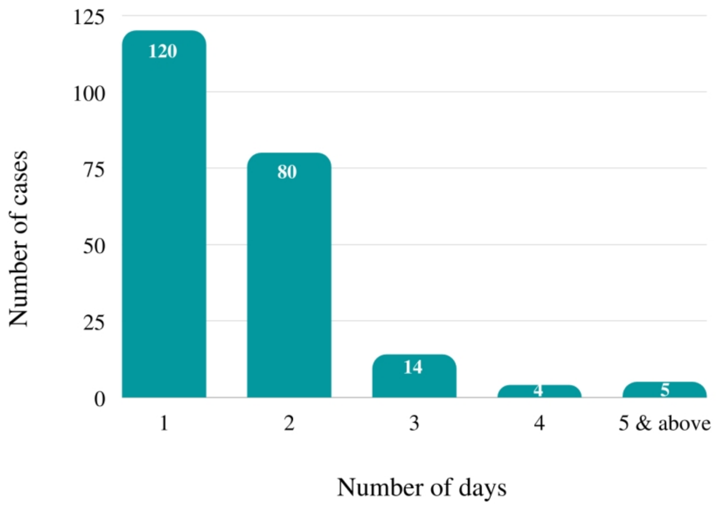


413x269mm (118 x 118 DPI)
